# Emerging Plasmonic Nanomaterials for SERS-Based Disease Diagnostics: Innovations, Clinical Challenges, and AI Integration

**DOI:** 10.3390/molecules31132225

**Published:** 2026-06-24

**Authors:** Rabeea Razaq, Arslan Younas, Muhammad Azam Qamar, Ahmad Farhan, Aman Khalid, Amna Akhtar, Muntaha Anwar, Tania Shad, Zulfiqar Ahmad Rehan, Syed Imran Hassan

**Affiliations:** 1 Department of Chemistry, University of Agriculture Faisalabad, Faisalabad 38000, Pakistan; rabeearazaq@gmail.com (R.R.); arslanyounas1998@gmail.com (A.Y.); ahmadfarhanuaf@gmail.com (A.F.); muntahaanwaruaf12@gmail.com (M.A.); 2 Department of Chemistry, School of Science, University of Management and Technology, Lahore 54770, Pakistan; qamariub@gmail.com; 3 International Center for Interdisciplinary Research in Sciences, The University of Lahore, Lahore 54000, Pakistan; 4 School of Mechanical, Medical and Process Engineering, Faculty of Engineering, Queensland University of Technology, Brisbane, QLD 4000, Australia; aman.khalid@hdr.qut.edu.au; 5 Department of Energy Systems Engineering, University of Agriculture Faisalabad, Faisalabad 38000, Pakistan; arsalakhtar72@gmail.com; 6 Department of Zoology, Wildlife and Fisheries, University of Agriculture Faisalabad, Faisalabad 38000, Pakistan; taniashad123@gmail.com; 7 Department of Chemistry, College of Science, Sultan Qaboos University, Al Khod, Muscat 123, Oman; z.rehan@squ.edu.om

**Keywords:** surface-enhanced raman spectroscopy, artificial intelligence, personalized medicine, early disease detection, biomedical diagnostics

## Abstract

Surface-enhanced Raman spectroscopy (SERS) has emerged as a transformative tool in biomedical diagnostics, offering a highly sensitive and non-invasive method for detecting molecular biomarkers at exceptionally low concentrations. This approach takes advantage of the plasmonic characteristics of customized metallic nanostructures that produce intense localized electromagnetic fields via localized surface plasmon resonance and facilitate electron transfer reactions that notoriously enhance the intrinsically weak Raman scattering signals of molecular entities which reside on or next to their surfaces. SERS-based assays have shown remarkable potential in detecting cancer biomarkers, circulating tumor DNA (ctDNA), and proteins at early stages, enabling timely and targeted intervention. Additionally, the combination of SERS with AI-driven data analysis has facilitated real-time diagnostics, enhancing the precision and efficiency of point-of-care testing. Despite its promising capabilities, challenges such as substrate fouling, signal degradation, and the need for better biocompatibility remain. Nevertheless, ongoing research in substrate development, coupled with advances in AI, positions SERS as a leading technology for future diagnostic tools. This paper explores the current state of SERS in biomedical applications, highlighting its potential to revolutionize diagnostics and personalized medicine while addressing the existing limitations and future research directions.

## 1. Introduction

Surface-enhanced Raman spectroscopy (SERS) has emerged as one of the most promising analytical techniques in the field of biomedical diagnostics. By exploiting the plasmonic properties of metallic nanostructures, SERS significantly enhances the Raman scattering of molecules, providing an extremely sensitive method for detecting molecular markers even at low concentrations [1]. This enhanced sensitivity allows for the detection of biomarkers that are critical for early disease diagnosis, making SERS an invaluable tool for personalized medicine and point-of-care diagnostics [2]. Over the past decade, technological advancements in SERS, such as improved substrates, nanostructures, and the integration with artificial intelligence (AI), have broadened its applications in medical diagnostics, including cancer, neurodegenerative diseases, and infectious diseases [3].

The incorporation of AI and ML into SERS technology has been a game-changer, completely altering the way data are analyzed and understood [4,5]. Because of the complexity of the data produced by SERS experiments, sophisticated computational approaches are necessary for their correct interpretation [3]. AI algorithms, particularly those based on machine learning, can now process SERS spectra to identify disease biomarkers with high accuracy. For example, in cancer diagnosis, AI-driven SERS systems are being developed to detect specific tumor markers in blood samples, offering a non-invasive alternative to traditional biopsy methods [3,6]. This combination of SERS with AI not only increases diagnostic precision but also facilitates real-time analysis, which is crucial for point-of-care applications.

Although the potential for SERS is advanced significantly, its clinical application has been lagging due to many challenges. The basic limitation is still substrate fouling: in the context of complex biological matrices, proteins and other biomolecules quickly adsorb on top of plasmonic surfaces to create a passivating layer encumbering the hot spot’s cell, significantly changing local dielectric environments, leading to progressive signal drift and (eventually) loss of reproducibility [7]. Discussing the increasing importance of long-term analytical performance in biofluids over quantitative analyses recently, matrix effects can clearly diminish it. Consequently, this has urged researchers to bestow antifouling strategies including zwitterionic polymer coatings for generating hydration barriers combating non-specific adsorption with elaborate sample pretreatment protocols and surface modifications maintaining enhancement as well as increasing the robustness in clinical matrices [8,9].

However, beyond clinical diagnostics, SERS has also quickly matured as an approach for diverse high-value commercial and field-deployable applications in non-medical sectors [10]. For food safety and agricultural quality control, SERS platforms now provide ultrasensitive detection of pesticide residues, mycotoxins, foodborne pathogens, adulterants, and chemical contaminants directly in complex real matrices including cereals, dairy, and animal-derived products [10]. Notable developments from the recent years are hybrid substrates incorporating magnetic nanoparticles for fast preconcentration and cleanup, graphene oxide components to increase chemical enhancement via favorable π–π interactions, gel-embedded nanostructures that imbue better stability and batch reproducibility as well as biorecognition elements [aptamers or antibodies] to promote enhanced selectivity in the presence of interfering food matrix components [11]. Such advances are raising standards for food safety monitoring and supply-chain verification worldwide, enabling faster than typical days or weeks waiting for test results that must go to a laboratory [12].

Together, these applications represent a clear progression from laboratory-based research towards durable, portable SERS systems that will be commercially useful in the field [13,14]. Enabling innovations include flexible and paper-based substrates that reduce costs and promote simple field-of-use, multimodal sensor designs that link SERS to complementary readouts, magnetic enrichment strategies, and artificial intelligence/machine learning interfaces for automating spectral interpretation/pattern recognition/kLaid analyte identifications in noisy field data [15,16]. Such advances are particularly significant in bottom-of-the-pyramid (BOP) contexts that would ideally be better serviced using affordable, rugged, decentralized platforms capable of performing food safety inspections, environmental surveillance, agricultural quality assurance, and security screening purposes completely outside of central laboratory infrastructure [17]. This leads to a vibrant ecosystem with SERS transitioning from a niche research tool to practical, point-of-need analytical technology in global food systems, environmental protection, and public safety domains [17].

Likewise, incorporating SERS in therapeutic drug monitoring (TDM) presents a new way of evaluating the concentration of drugs in patients in real time [18]. The use of SERS for the rapid field testing of hazardous substances, such as mycotoxins, confirms the technology’s ability to perform efficient field testing [19]. All the applications mentioned above show that there is a significant shift in the focus from lab-based research to the use of portable, handheld diagnostics and commercial monitoring systems in real bottom-of-the-pyramid (BOP) environments in health care and the environment [20].

This review is organized around a narrative describing the design of new plasmonic nanostructures and the tailored specific complex diagnostic applications of their particular physicochemical attributes, the electromagnetic enhancement features of Ag–Cu alloys or the chiral discrimination of bimetallic octapods. We analyze the synthesis of materials and their potential clinical applications and identify barriers to the medical application of these materials. Disease diagnosis has emerged as a booming application field for SERS-based detection due to its high accuracy and sensitivity. Recent studies have highlighted transformative advancements in this area; for instance, new diagnostic frameworks have been proposed for more precise clinical outcomes [21]. Breakthroughs in substrate engineering continue to enhance the detection limits for complex biomarkers [22]. Furthermore, the integration of advanced optical configurations and next-generation plasmonic materials is pushing the boundaries of photonic sensing in biomedicine [23], leading toward highly standardized and efficient diagnostic platforms expected to dominate the field in the coming years [24].

## 2. SERS

**Figure 1:**
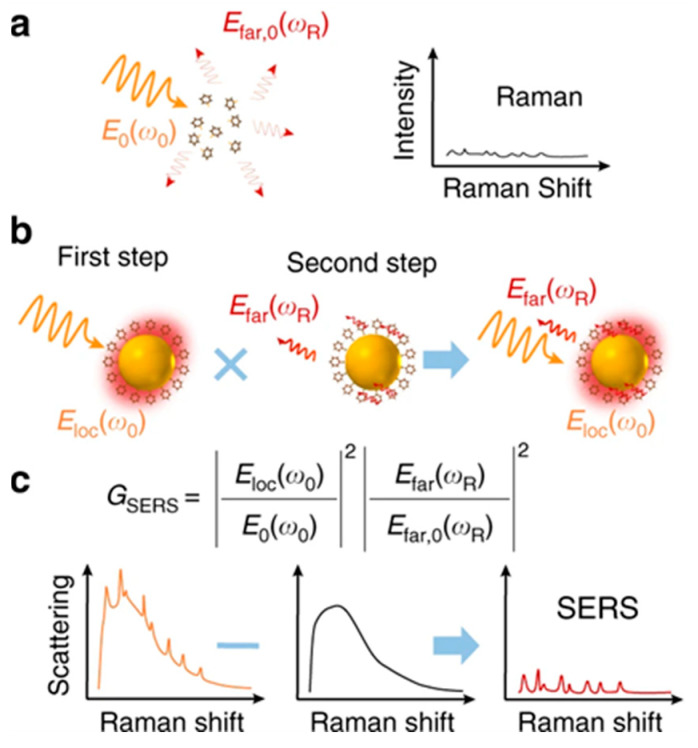
(**a**) Normal Raman scattering and Raman spectrum of molecules illuminated by narrow band laser. (**b**) Surface-enhanced Raman scattering from molecules adsorbed onto the metal nanosphere. (**c**) Data processing for SERS spectrum. Reproduced from [32] licensed under CC BY 4.0.

Interactions between light and molecules cause a change in the energy of laser photons, which results in Raman scattering, an inelastic kind of scattering. Structural information about molecules may be “fingerprinted” using Raman spectroscopy [25]. On the other hand, this spectroscopic method has a sensitivity problem because of the very weak Raman scattering. Raman scattering with a surface augmentation may significantly increase the strength of the Raman signal (Figure 1a). Analytes’ Raman signals may be amplified in the form of well-liked nanostructured materials such as nanoparticles (NPs), roughened films, or substrates with nano motifs. This is achieved by chemical contributions or surface plasmon augmentation. The target molecules may be quickly and non-invasively detected in situ using surface-enhanced Raman spectroscopy [26]. Because of its exceptional selectivity and sensitivity, the molecular resonance Raman Effect in conjunction with surface-enhanced Raman scattering is becoming more and more popular. Environmental analysis, food science, biomedicine, nanotechnology, biology, and surface and interface chemistry and related fields have all benefited from advances in surface plasmon resonance (SERS) theory, SERS substrates, and associated instrumentation [27]. Electromagnetic amplification under the influence of localized surface plasmon resonance and the creation of intense electromagnetic hot spots has now explicitly described Raman intensity scaling as |E|^4^ [28]. The complementary chemical mechanism (CM), or charge-transfer (CT) amplification, requires direct molecular adsorption (<1 nm). Static or photo-induced CT between analyte orbitals and metal states (or Fermi level) alters molecular polarizability through orbital hybridization and Herzberg–Teller vibronic coupling. This selectively boosts Raman cross-sections of specific vibrational modes. CM is molecule-specific—dependent on binding atom, adsorption geometry, and electronic alignment—and usually contributes 10^1^–10^3^. It exhibits a pronounced “first-layer effect.” EM and CM cooperate hot-spot fields increase CT probability, while chemisorbed molecules in hot spots experience both contributions simultaneously. Total enhancement is not a simple product but emerges from coupled photonic, electronic, and vibrational degrees of freedom at the heterogeneous interface, plus distance-dependent quenching and orientation effects [29,30,31].

The molecule can either be adsorbed or found/placed in the immediate vicinity of the substrate at a specific distance to be detected. Subsequent to the first identification of SERS on a textured silver electrode, several theories were proposed, grounded on electromagnetic and chemical principles, to elucidate the fundamental mechanisms of Raman scattering enhancement [33]. The mechanisms of enhancement have been extensively debated over the last many decades, resulting in the emergence of two predominant schools of thought as the most plausible. The essential role of surface plasmon in SERS has been shown. Light whose incidence wavelength is less than the particle’s size interacts with surface plasmons in a localized manner, stimulating them [34]. Oscillation frequency in relation to restoring force between nuclei and electrons is determined by the particle’s intrinsic features, which include its size, shape, and structure. When the light frequency is in phase with the electron oscillation frequency, an amplified electric field is produced at the surface of the particle in localized surface plasmon resonance. The Raman scattering in adsorbates is amplified by the electromagnetic field [34].

Materials exhibiting surface plasmon resonance absorption at wavelengths far removed from surface-enhanced Raman scattering (SERS) require substantial chemical contributions at frequently used laser excitation wavelengths (Figure 1a–c). Chemical enhancement theory postulates that charge transfer aids SERS [35]. Unlike the amplification of overall Raman bands by electromagnetic means, specific enhancements of Raman bands attributed to charge transfer are observable. The metal-to-molecule or molecule-to-metal pathway may induce the charge transfer transition (µCT), the orientation of which is significantly affected by the material, the molecule, and the laser intensity. Certain molecules may adhere to metal surfaces by the exchange of photo-induced electrons with the metal’s Fermi level, while some molecules may have electrons moved between the metal’s Fermi level and its lowest unoccupied molecular orbital whereas others may not [36]. The energy difference between semiconducting materials’ full valence band and unoccupied conduction band is analogous to the Fermi level of plasmonic nanoparticles in charge transfer processes. Instead of being exclusive, the two processes operate concurrently to generate SERS signals. When CT and Plasmon’s collaborate inside a metal-semiconductor heterostructure, they may generate unprecedented SERS signals [36].

The most powerful electromagnetic enhancement occurs in highly localized nanoscale regions known as “hot spots.” Hot spots are formed when incident light excites localized surface plasmon resonance (LSPR) in plasmonic nanostructures (primarily Au and Ag) [37]. The electromagnetic field is intensely concentrated in these regions, and because Raman intensity scales approximately with the fourth power of the local electric field (|E|^4^), even moderate field enhancements produce enormous signal amplification [38].

Armed with this mechanistic framework, attention naturally turns to the materials that actually realize these enhancement effects in practice. The effectiveness of SERS in biomedical settings is ultimately dictated by the physicochemical properties of the substrate, which must simultaneously maximize hot-spot density, ensure stability in complex biofluids, and maintain biocompatibility requirements that have driven the evolution from simple colloidal nanoparticles to sophisticated hybrid architectures.

## 3. ERS Substrates in Biomedical SERS

**Figure 2:**
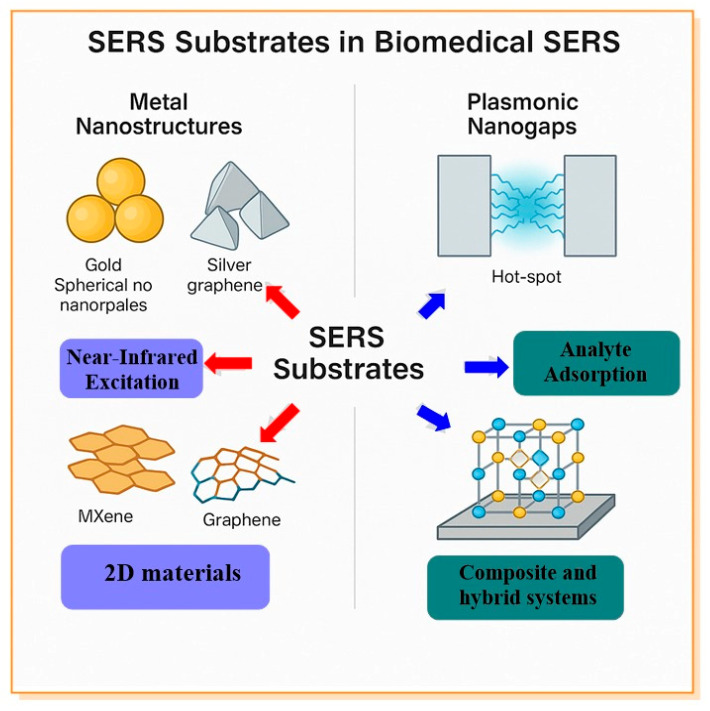
Evolution of SERS substrates in biomedicine. The schematic illustrates the transition from simple colloidal nanoparticles to advanced 2D/3D arrays and hybrid interfaces, highlighting design improvements to maximize “hot spots” and signal reproducibility for disease diagnosis.

The biomedical application of SERS is reliant on the specific physicochemical properties of the substrate. Recent innovations have gone beyond the simple use of spherical nanoparticles to more sophisticated, and in some cases, intricate designs. Here, the focus is on three main classes of such substrates: (1) noble metals (Pt, Pd, Au, Ag) are associated with high enhancement factors, albeit at varying costs and stabilities [39]; (2) copper-based substrates are cheaper, though prone to oxidation; and (3) hybrid nanostructures, which exploit the plasmonic power of metals and the biocompatibility of semiconductors or polymers [40,41]. The next subsections highlight certain unique characteristics for each of these materials in relation to clinical diagnostics [42]. The plasmonic properties of SERS substrates largely determine SERS’s effectiveness in biological investigation (Figure 2).

Therefore, to progress SERS technology, it is necessary to develop, study, and manufacture high-performance SERS-active nanostructures. The ideal SERS substrate would be long lasting, reproducible, and biocompatible. Limitations in functionality, lack of target selectivity, and complex matrix interferences are all issues with biological SERS that must be addressed [43]. There have been many stages of increasing complexity in the development of SERS substrates. Many other types of materials and structures have been explored for potential use as SERS substrates. Different types of substrates for SERS in biomedical application have been summarized in Table 1. These include semiconductors, composite nanostructures, two-dimensional materials, and metals with one or more components [44]. To avoid degradation of analyses and interference from fluorescence backgrounds, the SERS substrate should be designed to be excited by red near-infrared lasers (e.g., 633, 785, or 830 nm) in biomedical applications, especially for visible-speckled molecules or those that are photodegradable [45]. Moreover, these wavelengths correspond to the localized surface Plasmon resonances of all coinage metals used in SERS substrate preparation, which allows lasers operating at these frequencies to provide substantial signal enhancements [46].

The versatility and simplicity of fabricating metallic nanostructures from gold and silver have made them very popular. It is possible to modify the properties of these nanostructures by shaping them into various shapes and sizes. Solid SERS substrates may also be made by depositing them onto solid supports using various bottom-up techniques. Building larger systems from smaller ones, whether atoms, molecules, polymers, or nanoparticles, is what bottom-up synthesis is all about [47]. The bottom-up method makes use of methods including chemical synthesis, optical printing, template-based operations, and self-assembly [48,49,50,51].

By doing away with the need for expensive equipment or specialized cleanrooms, this synthesis approach makes the process more accessible and affordable. To achieve denser hot spots relevant to SERS, they also provide precise control over form and composition. Despite these advantages, they may vary between labs, have limited scalability, and may need multi-step synthesis techniques [52]. Thus far, the most popular choice for SERS-based applications has been spherical nanoparticles made of silver and gold. Nanorods, nanostars, nanocubes, and nanotriangles, all of which have sharp edges or corners, are examples of the anisotropic nanostructures that have been produced. Because of their plasmonic properties, these metal nanoparticles are subjected to a laser with the right wavelength, they might produce powerful localized electromagnetic fields. The Raman scattering intensity for molecules situated in the nanoscale gaps (“hot spots”) created by conglomerations of nanoparticles is amplified when these particles clump together. You may manipulate their plasmonic characteristics by adjusting their size and shape, and you can take advantage of extra resonance Raman amplification or decrease fluorescence background by using various visible and near-infrared excitation wavelengths [42].

To precisely manage the enhancement of Raman signals, plasmonic nanostructures with nanogaps that produce hot spots, nanogratings, and nanogrooves are painstakingly created. In order to provide a smooth and organized surface, the structures are built using top-down approaches. Photolithography, electron beam lithography, and focused ion beam lithography are all examples of such methods. The analyte’s collection and location inside the plasmonic nanogaps is a major challenge with these substrates. A comprehensive evaluation of the procedures for fabricating sub-10 nm gap-engineered substrates and for introducing analytes into these gaps was conducted [53,54].

The substantial surface area, molecule adsorption capabilities, and capacity to enhance charge transfer between the analyte and substrate of inorganic compounds like MXene and graphene have garnered interest, along with semiconductor materials such as TiO_2_ and ZnO [55]. To include repeatable and stable bespoke SERS applications, these materials with well-characterized electrical characteristics were used to functionalize plasmonic nanostructures with sophisticated optical features. MOFs, which are a combination of organic and inorganic materials, are useful substrates because they allow precise regulation of porosity, metallic node layout, and functionalization [56]. Improved analyte adsorption, cost-effective manufacture, longer shelf life, repeatability, and reusability are some of the issues that these new substrates have the potential to fix using SERS. They are able to maximize plasmonic enhancement and charge transfer.

Analytes of interest in evaluating SERS for biomedical applications might include a wide variety of substances, such as drugs, proteins, nucleic acids, bacteria, and viruses. The target analyte’s specificity dictates the SERS substrates and excitation wavelengths that must be used. Because larger macromolecules or cells cannot reach the nanogap, a substrate with a nanogap is not ideal for their analysis. However, a nanogap-containing substrate is better for detecting small compounds like metabolites or medications [56]. Colloidal SERS substrates are perfect for bigger macromolecules or cells when imaging using techniques like cell and tissue imaging. However, there are a few drawbacks to using colloidal substrates. One of them is the possibility of uneven hot spot distribution. Another is the uncontrolled aggregation. Lastly, the signal produced has limited repeatability in strength.

**Table 1:** Type of substrate for SERS in biomedical application.

Substrate Type	Detection Limits (Molar)	Sensitivity	Biomolecule Detection Applications	Material Properties	Main Applications	Challenges	References
Silver Triangle Nanoplates on PVC/SEBS Membrane	5.47 × 10^−8^ M	High	Cortisol	Flexible, high specificity, high sensitivity	Wearable detection, stress analysis	Fabrication complexity, sensitivity balance	[57]
Chitosan-Silver Nanoparticle Hybrid 3D Porous Structure	5 pM	High	Atopic Dermatitis Genetic Markers (Chemokines)	Low cost, high selectivity	Biomedical diagnostics, genetic marker detection	Long-term stability	[58]
Femtosecond Laser Processed Ag SERS Substrate	10^−8^ M	Very High	Food Safety, Environmental Detection	Laser-induced nanostructures, high uniformity	Food safety, environmental monitoring	Oxidation stability, uniformity	[59]
Ag NPs on CVD Graphene/Cu Foil	1 × 10^−14^ M	Very High	Rhodamine 6G (R6G)	Plasmonic coupling, high sensitivity	Environmental protection, high sensitivity detection	Fabrication cost, material instability	[60]
Flexible Multicavity AgNPs@NFA/PDMS Substrate	7.4 × 10^−13^ M	High	Thiram, Pesticide Residues	High mechanical stability, flexible, high sensitivity	Food safety, agricultural monitoring	Fabrication complexity, sensitivity under real-world conditions	[61]
Bimetallic Ag-Cu Microflowers	1 fM	Ultra-High	Anticancer drugs (Mitoxantrone, DOX)	Superior electrical conductivity; high hot-spot density	Therapeutic drug monitoring	Surface oxidation of Cu component	[62]
MXene-Ti_3_C_2_T_x_/Ag NPs Hybrid	10^−14^ M	Ultra-High	Mir-21 (Cancer biomarker)	High surface area; Metallic-like conductivity	Early cancer diagnosis; Liquid biopsy	Synthesis complexity; Long-term stability	[63]
Noble Metal Nanoparticles	10^−9^ to 10^−14^ M	Very High	Proteins, DNA, PSA	High LSPR, Au/Ag based	Cancer Biomarkers, Viral detection	Poor reproducibility, oxidation of Ag	[64,65]
2D Hybrid Materials	10^−12^ to 10^−15^ M	Ultra-High	ctDNA, Exosomes, Cancer cells	MXene (Ti_3_C_2_T_x_), Graphene Oxide	Early-stage Liquid Biopsy	Complex synthesis, stability in bio-fluids	[66]
Semiconductor Oxides	10^−3^ to 10^−6^ M	Moderate	Glucose, Cholesterol	ZnO, TiO_2_, Cu_2_O	Diabetes monitoring, Metabolic disorders	Lower Enhancement Factor (EF)	[67]
Flexible/Paper Substrates	10^−7^ to 10^−9^ M	High	Pesticides, Viral RNA (COVID-19)	Cellulose/AuNPs, Polymer films	Point-of-Care (POC) Diagnostics	Signal background from substrate	[68,69]
Multimetallic Core–Shell	10^−10^ to 10^−13^ M	High	Cardiac Troponin, MicroRNA	Au-Ag alloys, Au@Pd	Myocardial infarction diagnosis	Precise shell thickness control	[70]

### 3.1. Noble Metals as SERS Substrates, Pt/Pd

Among the many notable electrochemical and catalytic capabilities of the noble transition metals palladium and platinum are their widespread use in photocatalysis and electrocatalysis [71,72]. Because surface plasmon resonance is quenched by interband excitation in the visible spectrum, these metals are usually considered inactive substrates [73]. One of the main reasons why surface-enhanced Raman spectroscopy is able to produce stronger signals than regular Raman spectroscopy is because of surface plasmon resonance. To improve the efficacy of SERS, surfaces made of palladium or platinum may be electrochemically textured [74].

Tian et al. have made important contributions to this field by conducting a groundbreaking work that used roughened platinum for SERS measurements. Pt and Pd are traditionally considered less active for SERS due to interband damping; their electrochemical stability makes them ideal for monitoring biomarkers in acidic or corrosive clinical environments. For instance, this stability is paramount for the reliable detection of Alzheimer’s biomarkers in clinical cerebrospinal fluid (CSF) samples, where high reproducibility is required for early diagnosis. By preventing the quenching of surface plasmon resonance, the borrowing effect has the ability to enhance the SERS activity of Pt and Pd by encasing Au and Ag-based active metal cores in Pt and Pd shells. This research demonstrates that SERS may be applied to a far greater variety of materials and surfaces by efficiently synthesizing nanostructures, making it a more powerful diagnostic tool [75].

**Figure 3:**
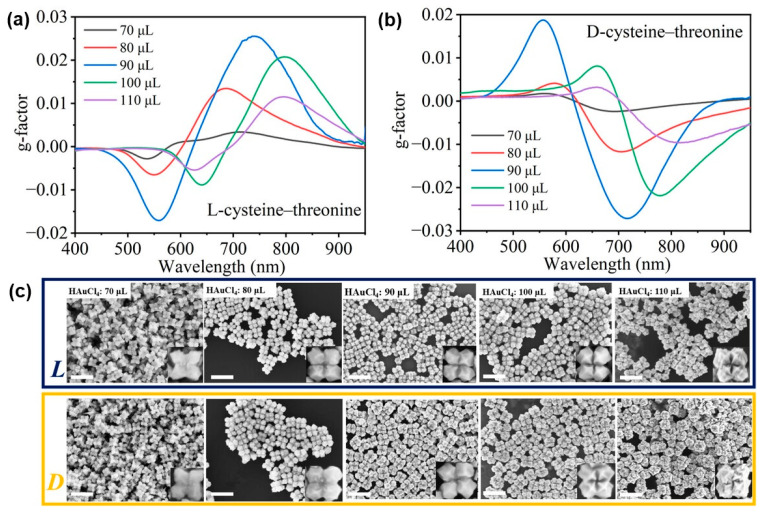
(**a**) CD spectra of chiral Pt@Au octapods produced with differing quantities of HAuCl_4_, using (**a**) l-CT and (**b**) d-CT as chiral ligands. (**c**) SEM pictures of chiral Pt@Au octapods demonstrate the influence of HAuCl_4_ quantities on the morphological modulation of chiral Pt@Au octapods. Reproduced with permission from [76].

**Figure 4:**
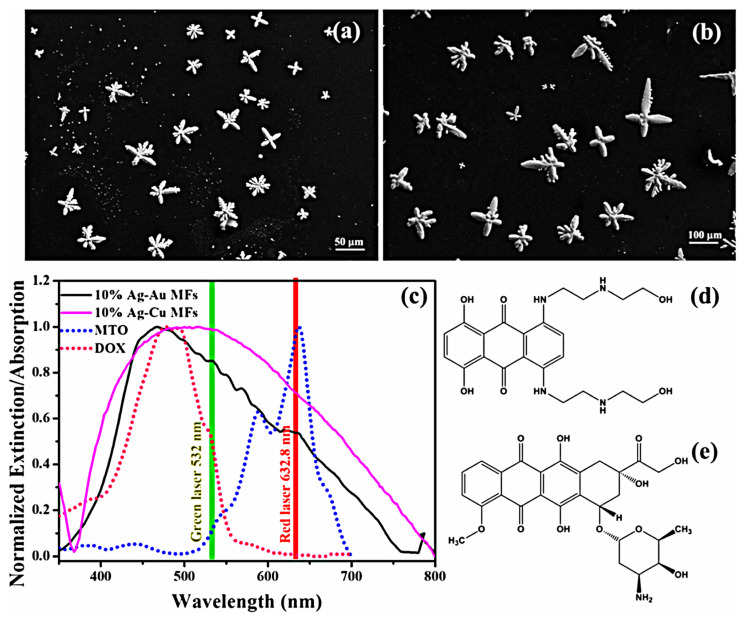
Comparative analysis of bimetallic SERS substrates. (**a**,**b**) FE-SEM images showing the morphology of Ag-Cu and Ag-Au microflowers. (**c**) Extinction spectra demonstrating the superior electromagnetic enhancement of Ag-Cu alloys for the multiplexed detection of anticancer drugs MTO and DOX. (**d**,**e**) Molecular structures of these drugs. Reproduced with permission from [62].

In their study, Li and colleagues successfully synthesized chiral bimetallic Pt@Au octapods using l/d-cysteine–threonine (CT) dipeptide as chiral ligands. These octapods had a unique structure, resembling a spiral four-petal flower with a twisted concave shape on each facet. The concave design played a crucial role by enhancing intraparticle interactions, creating concentrated electric fields in the concave regions. The result was the development of “hot spots,” which amplified the SERS effect to a far greater degree. The l-Pt@Au octapods exhibited nearly double the SERS response to Aβ40 and Aβ42 monomers and fibrils compared to their d-Pt@Au counterparts. This difference was attributed to the distinct chiral recognition ability of the CT ligands on the surface of the l-Pt@Au octapods (Figure 3a,b). These ligands formed specific hydrogen bonds with the Aβ40 and Aβ42 monomers and fibrils, which in turn caused noticeable variations in their Raman spectra. Further analysis of clinical cerebrospinal fluid (CSF) samples revealed that the Aβ42/Aβ40 ratio, measured by Raman spectroscopy, could serve as a reliable biomarker for the early detection of Alzheimer’s disease (AD), with a cut-off value of 0.085. This breakthrough opens the door to using chiral nanomaterials with strong optical properties for the development of advanced clinical diagnostic tools in the biomedical field [76,77]. The SERS spectra in Figure 4c show multiplexed identification of anticancer drugs Mitoxantrone (MTO) and Doxorubicin (DOX). These spectra show differences in the distinct peak positions and intensities of the Ag-Au and Ag-Cu substrates. These differences are attributed to the following:

The position of MTO and DOX molecules relative to the “microflower” (MF) surface controls the extent to which particular vibrational modes are amplified [70]. The Ag-Cu alloys exhibit a more extensive extinction profile in the red-laser range (632.8 nm), which is more preferable to MTO molecular resonance, deriving more intense peaks than Ag-Au [62]. The minute difference in charge-transfer levels between the bimetallic surface and the drug functional groups results in the disparity in the Raman scattering cross-sections.

Among several noble metals, gold (Au) and silver (Ag) stand out as the most dominant for usage in SERS because of their unique electromagnetic enhancement. However, other noble metals such as platinum (Pt), palladium (Pd), and copper (Cu) also present unique practical benefits in disease diagnosis. In spite of possessing lower enhancement factors, platinum and palladium substrates, present for oxidation and other reactions, catalytically respond with greater efficiency. Thus, they present substrates for the detection of redox-active biomarkers and other enzymatic reactions during acute or variably acidic microenvironments of a tumor, in which silver substrates would present rapid oxidation and degradation. In addition, copper (Cu) is now being recognized as a more affordable option for Therapeutic Drug Monitoring (TDM). Recent investigations and analyses have revealed that well-stabilized copper nanostructures can be relied upon for SERS signatures which are associated with the detection of clinically relevant antibiotics and anti-cancer drugs, thus providing the most sustainable platforms for accessible large-scale diagnostic screening in developing countries.

It is likely that transition metals like palladium and platinum will be combined with conventional SERS substrates or metal alloys since these elements do not exhibit SERS activity when used alone [78,79]. The surface of the Si layer is often coated with Pt/Pd films or nanoparticles, while SERS studies seldom make use of uncoated Pt/Pd nanoparticles. The enhancement factor (EF) of the Pt and Pd substrate group is 1.4 × 10^5^ which is almost twice as low as the geometric average EF of Au/Ag and Si-based substrates. Substrates based on Pt/Pd have a detection limit of about 2.9 × 10^−9^ M, which is one hundred times lower than the limits of the aforementioned Au, Ag, and Si groups. Platinum and gold were among six metals including pyridine—documented by Tian et al. in 2002 as SERS signals [80]. The study found that compared to platinum, whose enhancement factor was 2000, gold’s EF of 10^6^ was about 500 times higher. Nanoparticles of other metals, such as Rh, Fe, Ni, and Co, showed an improvement on par with platinum’s.

In work conducted by Kim Kwan, 4-aminobenzenethiol was used to interpose silver nanoparticles onto Au or Pt surfaces, further demonstrating that gold is superior than platinum as a SERS substrate. While researchers could not find any SERS signals when testing 4-ABT on a Pt surface, they did find an increase of 790 when using AgNP in a sandwich format. The surface EF for Au was 1.7 × 10^4^ [81]. In a different experiment, p-ATP on a Pt film was examined using Raman spectroscopy, and no signal peaks were seen. The analyte was encapsulated using silver nanocubes, the scientists found that an optimal enhancement factor of 4.1 × 10^6^ is achieved, and that this enhancement factor almost triples when the Pt film thickness grows from 42 nm to 90 nm.

A fifteen-fold improvement over the glass surface was seen when using the platinum substrate in comparison to the AgNC/p-ATP@glass surface substrate in the same study [78]. The microfluidic channels were enhanced with Ag-Pd alloy nanostructures by means of the technique for direct writing using femtosecond SERS. A substrate integrated into a microfluidic channel, chemical reactions may be monitored in situ and detected on-chip. Microfluidic technology may have also improved the SERS process, allowing for analyte concentrations as low as 1 nM to be detected. In addition, the Ag-Pd alloy helped make it resistant to aerobic oxidation [82].

### 3.2. Copper

Because of their inexpensive price, high SERS activity, and great ability to chelate most analytes, copper-based substrates hold great promise for SERS studies [83,84]. Copper has more applications because, like gold and silver, it sustains surface plasmons in the visible-to-near-infrared (NIR) region, rather than in the UV band [85]. Even with surface oxidation problems, copper substrates possess certain diagnostic benefits, most notably for large-scale bacterial screening and therapeutic drug monitoring (TDM) [86]. Unlike silver and gold, copper’s ability to support surface plasmons in the UV band permits the resonance and detection of certain metabolites and small molecules that do not resonate with conventional red or NIR laser excitation. Given this unique optical property, in combination with its low cost, copper-based platforms can be a leading alternative for large-scale analytics in clinical settings [87].

Compared to silver and gold, it is not used as often in plasmonic applications because of instability and surface oxidation [88]. Copper may be protected against surface oxidation in a variety of ways. Under Dr. Van Duyne’s direction, researchers discovered that glacial acetic acid efficiently dissolves copper oxides (Cu_2_O and CuO) without causing any surface changes to the metal. They proved that when the oxide layer is removed, the localized surface plasmon resonance peak becomes somewhat thinner and more intense, similar to what happens with gold and silver when λ_max_ is greater than about 650 nm [88].

When it came to making SERS substrates out of copper, Dr. Van Duyne’s lab was one of the first [89]. The majority of SERS-based investigations conducted in the 1980s made use of copper electrodes. In 1980, Dr. Van Duyne’s group discovered a substantial correlation between copper’s optical characteristics and surface-enhanced Raman scattering of pyridine on surfaces of copper electrodes as a function of wavelength. An excitation wavelength of 645 nm yields an enhancement factor of 6 × 10^4^ for the pyridine/Cu combination. When it came time for SERS active substrates, Cu island films and alloys were much sought after [89]. In their study, Kudelski et al. conducted in situ SERS investigation of adsorbed pyridine using amorphous Cu-Zr alloys. Their research suggests that by looking into the possible dependence of SERS intensity, it is possible to detect different clusters of copper on the surface of the substrate [90].

Copper’s integration with nanostructured silver or gold shows potential for further enhancement of SERS stability and sensitivity. For example, silver nanostructures bonded to copper-based substrates can increase the enhancement factor by an order of magnitude, from 10^6^ to 10^7^. Furthermore, the limit of detection (LOD) can be improved from 10^−9^ M to 10^−12^ M. This bimetallic approach proves to be advantageous with regards to the so-called “silver shell” protective mechanism, which helps maintain SERS intensity. This mechanism is especially useful with copper chips, which tend to have little SERS intensity to begin with, after a period of time. While pure silver chips with SERS sensitivity lose about 90% of their SERS intensity, copper with the silver shell and SERS can maintain 80% of the original SERS intensity even after two months of storage [91,92].

Copper structures of varying sizes and shapes are currently among the substrates that are active for surface-enhanced Raman scattering, including nanoparticles, Cu-coated fabric, copper triangular plates, films, and Cu nanowires.

Copper has tremendous promise as a material for the creation of SERS platforms utilized in biosensing [93,94,95,96,97]. It is economically favorable, displays higher physical stability over time than silver, and might show elevated enhancement factors between 10^6^ and 10^7^, this is similar to the (EF 10^7–10^) and (EF ~10^6^) values for silver and gold, respectively [98,99]. Copper SERS systems are effective for bacterial detection. An innovative copper-based platform was created by decomposing copper hydride using a fundamental high-pressure method. Similar to platforms based on silver and gold, the platform showed a significant improvement in S. aureus bacterial bands [98].

In order to determine how well different compositions of Composite Melamine Formaldehyde SERS substrates made of Ag-Au and Ag-Cu detected the crucial anticancer medication Mitoxantrone, Kaja et al. examined their performance (Figure 4a–c). The 10% Ag-Cu substrate was shown to be better in the SERS tests, which used two different excitation laser sources (532 nm and 632.8 nm). Following activation with the 632.8 nm laser, the 10% Ag-Cu substrate achieved better results than the 10% Ag-Au substrate, fulfilling the criteria for surface-enhanced resonance Raman scattering in Mitoxantrone detection. The lowest limit of detection for Mitoxantrone ever reported was 1 fM using the 10% Ag-Cu sensor, setting a new standard for analytical methods. Research using theoretical COMSOL simulations of the related plasmonic structures further confirmed that the Ag-Cu substrate outperformed the Ag-Au substrate (Figure 4d,e). Consistent with the experimental findings, the electric field enhancement maps produced by the simulations showed that the Ag-Cu substrates exhibited a higher electromagnetic field enhancement. The enhanced performance could be because Cu has a better electrical conductivity than Au. In addition, for the detecting Mitoxantrone and another anticancer drug, doxorubicin, in both water and mouse blood plasma, the 10% Ag-Cu alloy substrates demonstrated excellent multiplexing capabilities. For Mitoxantrone, the LOD value was 1 pM, and for DOX, it was 10 nM, when the drug combination was spiked straight into the blood plasma without dilution. Such SERS sensors are expected to find widespread usage in clinical settings as miniaturized Raman spectrometers continue to improve [62].

Lastly, it should be noted that copper sulfide nanoparticles were used effectively in the development of SERS probes, which have garnered considerable interest as of late because to the promising biological applications they provide. There is still some trace of non-degradable Au or Ag nanostructures in the imaging tissue from SERS probes that have been described so far. Chronic toxicity is a major worry due to the SERS probes’ potential to cluster, produce harmful chemicals, and redistribute to other important organs, such as the heart, liver, lungs, and kidneys, which is heightened by their longer endurance [100]. This limitation prevents their further usage in living organisms. Alternatively, this limitation is circumvented using SERS probes made of photodegradable hollow copper sulfide nanoparticles. Qui et al. recently shown that CuS nanoparticle SERS probes may self-clearance from tumor tissue by degrading into smaller particles and eliminating residual tumor lesions by heat [101].

They modeled the disease after orthotopic prostate cancer. The proportion of copper (Cu) that remains in the major organs was documented, in particular the heart (0.6%), liver (0.4%), lungs (0.9%), and kidneys (0.3%), on day 30, indicating the efficient self-clearance of the CuS SERS probes over the 30-day period [102]. Even more impressively, CuS probes showed 3.6% ID/g of Cu inside the tumor, indicating excellent targeting capabilities. These results demonstrate that the principal organs were successfully spared from CuS SERS probes, which further supports their biosafety. Another study comparing the biodegradability and toxicity of hollow gold nanospheres with copper sulfide nanoparticles recorded similar results [100]. This study’s results show that by day 30, all traces of CuS nanoparticles had been removed, and they had not shown any toxicity in the chemical evaluations of blood or histology.

On the flip side, long-term toxicity was shown by higher blood lactate dehydrogenase levels caused by the persistent deposition of Au NPs in the liver. The degradability of CuS NPs SERS probes, in comparison to those based on Au or Ag, allows for the use of SERS in many biological settings. The most significant analytical studies conducted on copper-based substrates doped with Ag or Au are described in detail here [103,104,105].

Dai et al. found in their investigation that pure Cu surfaces treated with Ag resulted in Cu chips with a 103-fold lower crystal violet limit of detection, from 10^−8^ M to 10^−11^ M. Also, the nanoscale plasmonic interaction between Cu and Ag explained the observed rise in the enhancement factor from 2.0 × 10^6^ to 7.6 × 10^6^ [103]. After two months in storage, the protective Ag shell allowed the intensity of Ag-modified Cu chips to retain 80% of its original value, whereas the original Cu chips only retained 18%. Compared to Ag nanoparticles alone, Fodjo et al. showed that a SERS substrate made of Ag/b-AgVO_3_ nanobelts on copper foil has better SERS activity for carbamate insecticides. The modified substrate was stable throughout time and showed remarkable repeatability across ten separate measurements of the different pesticides (RSD 4.3%) [105].

When Cu is added to the Ag-Cu alloy on various microflower substrates, the SERS intensity of the probe molecule R6G grows in a linear fashion as shown by Sravani et al., and reaches its maximum at 10% Cu compared to pure Ag MFs. Using 10% Ag-Cu alloy microflowers, they were able to achieve SERS spectra that were 50 times better than those produced with pure Au-Ag microflowers [106]. Presently, plasmonics, catalysis, and SERS are vying for bimetallic nanostructures that can be shaped to suit their needs [107]. Siva et al. demonstrated a significant SERS signal for crystal violet using Au−Cu NSs ranging in size from 200 to 300 nm. At concentrations as low as 10^−10^ M, they were successful in identifying CV molecules and attaining an enhancement factor of up to 10^6^. The concentration of Ag has a substantial impact on the modification of the Cu substrate, as shown by Rao et al. [108]. Only at a 12 mM concentration of Ag particles does the spectrum augmentation work well; at higher concentrations, the signal is reduced.

Biosensing applications in tumor and cancer cell detection have shown potential for novel copper alloys including noble metals as SERS substrates, in addition to excellent sensitivity and stability. For the purpose of detecting lung cancer cells, Wen et al. recently used a photoreduction approach to synthesis porous CuFeSe_2_/Au heterostructure nanospheres [109]. The CuFeSe_2_ nanocrystals were exposed to near-infrared light with a wavelength greater than 850 nm in order to create the nanospheres. A gold shell was then deposited onto their surface. In addition to high selectivity and sensitivity (1.0 ppb), by photodegrading undesired absorbed biomolecules, the produced substrate showed exceptional photocatalytic cleaning efficacy. This characteristic could be very important in biomedical and bioanalytical tests. Surface oxidation is a common problem with copper substrates, which is why they are not often employed in the plasmonic applications. Gold and silver, which are nanostructured noble metals, may be able to resolve this issue. The plasmonic characteristics of copper-based nanostructure seem to be superior to those of pure copper, according to the SERS study. This may be because the metals’ protective and enhancing capabilities make them more effective.

Sunil et al. created a biosensor for oral cancer mass screening that uses coated silver nanoparticles with an Ag core and a carbon nanofiber shell for very sensitive detection of salivary biomarkers. The surface-enhanced Raman spectroscopy is the foundation of the sensor. This SERS substrate is very exceptional, with a detection limit for rhodamine 6G molecules as low as 10^−12^ M and an incredible Raman signal enhancement of up to 10^7^ times. Studies using finite-difference time-domain simulations on Cu@Ag/CNFs found that the electric field intensity enhancement factor at the plasmonic hot spot formed between two nearby Cu@Ag nanoparticles was 250. The investigation also looked at the variations in Raman peaks in saliva from those with oral cancer compared to healthy controls. Distinct differences were observed between the two groups in their average SERS spectra. The data distributed as per principal component analysis (PCA) with two dimensions (PC-1 and PC-2), where PC-1 is aligned horizontally and PC-2 is aligned vertically, as a result of using scatter plots based on principal component analysis to compare the data. The study’s findings demonstrated a high level of precision (87.5%), high specificity (92%), high sensitivity (88%), and a low F-measure (88.5%). This means that the suggested PCA using the random forest approach successfully diagnosed malignant patients about 88% of the time. However, the test accurately classified 87.5% of patients, demonstrating a remarkable difference between the cancer and control groups [110].

Electrochemical interactions may greatly improve the surface plasmon resonance characteristics of the Au-loaded copper oxide nanoplates, according to both theoretical and spectroscopic studies. The drug molecules and the SERS substrate electrochemically interacted, leading to an enhancement of many Raman peaks related with hydroxycarbamide (HC), including NH_2_ wagging vibrational modes, NO stretching, CO stretching, CN stretching, and NCN bending. In ideal circumstances, a concentration as low as one tenth of a nanomolar may be detected. This methodology has the potential to be used in biomedical laboratories for quick and reliable assessment of HC, paving the way for its application in clinical diagnostics [111].

The EC-SERS enhancement for HC detection is, apart from the increased surface concentration, an effect of different considerations. The applied potential changes the SERS signal by shifting the Fermi level (EF) of the metal substrate (resonant) with the molecular orbitals of HC, which due to the severing of interfacial chemical bonds, results in an extensive charge transfer (chemical mechanism). The potential also changes the adsorption position of HC on the surface from “flat-on” to “vertical” (depending on peak adsorption), as dictated by potential-dependent adsorption isotherms. This shift brings important vibrational modes “closer” to the hot spots, thus optimizing the electromagnetic-chemical synergism [112].

**Figure 5:**
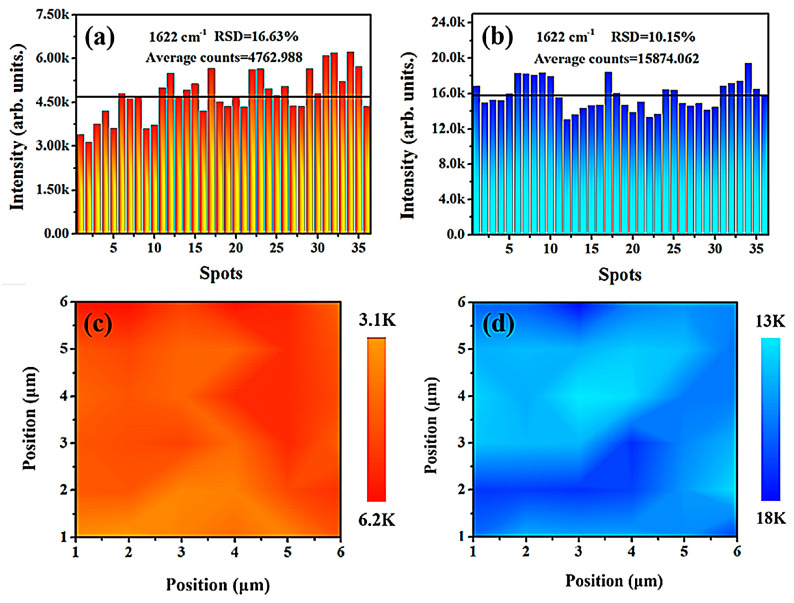
(**a**,**b**) The SERS intensity of 10^−5^ M MB at a typical peak of 1622 cm^−1^ was obtained from 36 randomly selected sites on the AgCu@MW-20 and Ag@Cu@MW-3 substrates. (**c**,**d**) SERS mapping image depicting the SERS intensity at the aforementioned 36 spots. Reproduced from [113] licensed under CC BY 4.0.

Song et al. demonstrated that bimetallic nanostructures, synthesized by varying the metal content ratio, may modulate the optical properties of the substrate. Furthermore, it was shown that the scale of moth wings (MW) including 3D grating-like uniform nanoarrays utilizing bioscaffold may consistently yield a high-density “hot spot” for the prepared plasmonic substrate. Two distinct approaches were utilized for the construction of SERS-active substrates: (i) co-sputtering for varying durations, and (ii) sequential alternating sputtering to create a layered structure on the MW; these methods were designated as AgCu@MW and Ag@Cu@MW, respectively. The comparative SERS measurement results of the two substrates utilizing probe molecules R6G and MB indicate that the stratified structure of the Ag@Cu@MW-3 substrate exhibits superior SERS performance (Figure 5a–d). This technique can generate plasma substrates with on-demand optical responses and offer novel concepts for the fabrication of traditional biomimetic nanomaterials [113].

### 3.3. Gold Nanoparticle

The surfaces may serve as a basis for Raman scattering with surface enhancement, which may be created by thiol ligands linked to gold nanoparticles. Beyond traditional citrate-capped gold nanoparticles, modern diagnostic research focuses on functionalized gold nanostars and chiral bimetallic octapods. These emerging materials provide high “hot-spot” density at their sharp tips, enabling the detection of specific DNA mutations (e.g., BRAF V600E) with 10 times higher sensitivity than conventional gel electrophoresis. For example, the use of chiral bimetallic Pt@Au octapods has shown specific recognition for Alzheimer’s disease biomarkers in clinical samples due to their unique morphological modulation [114]. The Raman internal standard is 4-mercaptopyridine, and the spacing between particles is controlled by dodecanethiol [115]. Frozen citrate-capped Au nanoparticles were then melted to produce Au nanoparticles with enhanced SERS activity [116]. A SERS signal was recorded indicating an increase from 1 to 6 arbitrary units (a.u.) in a controlled capillary assembly of gold nanoparticles in two-dimensional matrices [117]. Analytes’ Raman signals were boosted when cucurbituril spacers were used to create hot spots for surface-enhanced Raman spectroscopy using 0.9 nm gaps between Au nanoparticles [118]. An efficient SERS substrate is a two-dimensional superlattice made of poly(ethyleneglycol)methyl ether thiol-bound plasmonic clusters of gold nanospheres [49]. Optimizing chemical properties in surface-enhanced Raman spectroscopy, the presence of p-methylbenzenethiol on gold nanoparticles demonstrates Raman scattering mechanisms. Transforming citrate-stabilized Au nanoparticles into asymmetric Au nanoparticles using polystyrene-b-poly(4-vinylpyridine) resulted in a doubling of the SERS intensity [119]. The conversion of Au nanoparticles into mesoporous structures using a solution of polyvinylpyrrolidone and hydrofluoric acid allowed for the creation of the “hot spots” for SERS [120]. Outside the realm of engineering hot spots, functional SERS probes have been created for environmental monitoring, including the pH-sensitive nanosensor that uses SERS spectral changes of aromatic amine labels bonded to citrate-stabilized gold nanoparticles to identify alterations in surrounding pH levels [121]. This created “hot spots” for adjustable SERS by using femtosecond laser irradiation, annealing, and gold coating to link Au NRs to Au NPs [122].

Cluster formation mediated by the addition of 2-naphthalenethiol ligand formed the powerful electric field connecting Au nanoparticles [123]. An interface was created between a 1,2-dichloroethane and gold nanoparticle solution in order to build a plasmonic metal liquid SERS platform [124]. Prussian blue shell functionalization is applied to the citrate-stabilized Au nanoparticles coated with CN. Pb^2+^, Co^2+^, and Cu^2+^ form the crystal structure of this shell. One possible use for the Au@PBA nanoparticles in SERS is as a supermultiplex label [125]. After being treated with ethanol and thiolated polyethylene glycol to alter its surface charge density at the toluene–water interface, the evaporated Au NPs metafilm may be used as a SERS substrate for thiram detection [126]. There is evidence that the control of hot-spot formation for surface-enhanced Raman scattering (SERS) can be fine-tuned via the assembly of ligand-mediated conjugates. In addition to mere thiol–ligand interactions used for linking gold nanoparticles (AuNPs), more sophisticated systems can be used for fine control of the spacing and scattering of particles of the biotin–streptavidin recognition system. Previous studies integrating the effects of SPR and SERS demonstrated that biotin covalently linked to gold surfaces and STV-AuNPs had both selective and non-selective interactions. In the SPR sensor response, the binding of STV-Au nanoparticles to the biotin/11-mercapto-1-undecanol monolayer results in a response of about 0.15 RU, which leads to more than a 2-fold enhancement of the SERS response. SERS spectra of the biotinylated gold surfaces and STV-AuNPs at different time intervals confirmed that streptavidin and biotin interact during the SERS measurement at different time intervals. Additionally, the absence of biotin and the aggregation of STV-Au nanoparticles lead to non-specific contact with the gold screen, and multivariate curve resolution can be used to distinguish between these specific and non-specific interactions [127].

Researchers looked at 1,4-benzenedithiol’s SERS spectra using a sphere–plane sample that had a rough Au substrate in the middle and a configuration with Au nanoparticles on top. While both types of enhancement are chemically identical, sphere–plane samples exhibit better electromagnetic enhancement than Au rough surface samples [128]. A localized electric and magnetic field at the nanogap is produced when a single configuration of gold nanospheres and gold films (Au (III)) separated by mercapto benzoic acid are exposed to light. As a consequence of the interference between the two modes, this activates a magnetic dipolar mode and causes Fano resonance [129].

Low-energy ion-beam irradiation and oblique-angle gold deposition methods were used to create the gold nanoparticles within ripple templates. With increasing thickness of the gold sheet, plasma resonance shifts from 600 nm to the mid-infrared range. Through the use of 632.8 nm excitation and 120 nm thick Au nanoparticles, a maximum Raman enhancement factor (EF) of 1032 may be attained. The anisotropic SERS effect with improved polarization is seen in annealed Au nanoparticles, while the highest polarization increase in non-annealed Au nanoparticles is observed at 638 nm excitation. Annealed films containing Au nanoparticles exhibited SERS effects due to their wavelength and polarization-dependent hot spots [130].

With hydrogen peroxide present, the SERS substrate for rhodamine 6G and crystal violet research is the aggregated gold nanoparticles’ localized surface plasmon resonance hot spot. The gold nanoparticles’ citrate capping agent oxidizes when exposed to hydrogen peroxide, resulting in surface-enhanced Raman scattering hot spots. A decrease in colloidal stability increased the SERS signal intensity, whereas (i) dye molecular oxidation and (ii) degradation of existing hot spots were responsible for the attenuation of intensity. Using H_2_O_2_ lowers the detection limits for both rhodamine 6G dyes and crystal violet to 1  ×  10^−9^ M [131,132].

**Figure 6:**
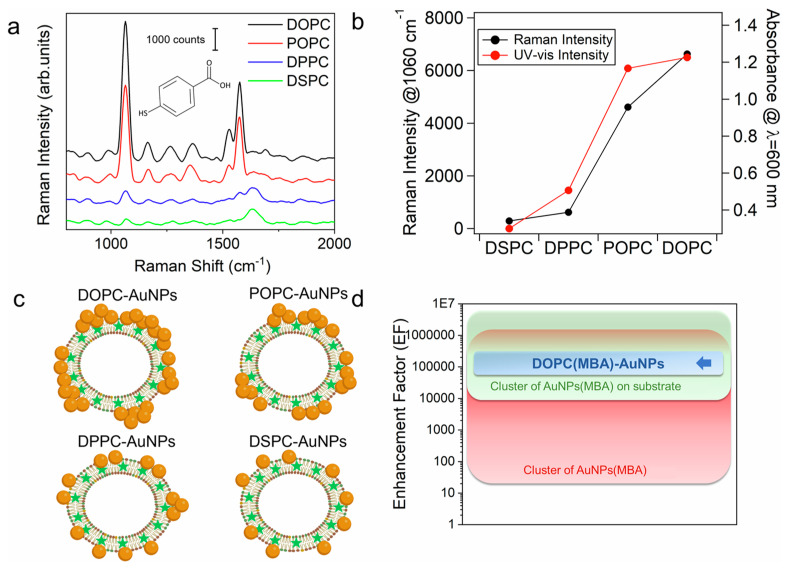
(**a**) Raman-SERS spectra obtained for several liposome types (DSPC, DPPC, POPC, and DOPC) including embedded 4-MBA molecules inside the bilayer. (**b**) Comparison of Raman intensity at 1060 cm^−1^ and UV–Vis signal at 600 nm. (**c**) Graphical depiction of the diverse interactions between AuNPs (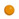) and several liposome types (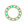). (**d**) Average enhancement factors for clusters of colloidal gold nanoparticles coupled with 4-mercaptobenzoic acid. Produced from [133] licensed under CC BY NC ND 4.0.

In their work, Cardellini et al. presented LipoGold Tags, a straightforward platform that allows clusters of gold nanoparticles to self-assemble on lipid vesicles. The increased electromagnetic field causes the reporters’ Raman signals to be magnified when they are put into the lipid bilayer. Researchers varied the proportions of lipid vesicles and Raman reporters to achieve full structural characterization and optimal surface-enhanced Raman scattering enhancement. The Raman reporters are anticipated to encounter the high electric field created by plasmon resonance near the Au surface, which results in an increased Raman signal due to the SERS effect (Figure 6a). Irrespective of the lipid vesicles’ very equal phospholipid contents, the Raman spectra obtained from each sample showed striking variations. The aggregation propensity of AuNPs was highly correlated with the MBA’s Raman intensity, as seen in (Figure 6b). The Raman intensity of both MBA peaks became stronger when the vesicle stiffness decreased (from DSPC to DOPC), indicating that the aggregation of nanoparticles on the lipid surface increased. The creation of additional “hot spots” within the AuNP clusters is the likely cause of this. These “hot spots,” or regions of strong local field amplification caused by plasmonic coupling, allowed the MBA Raman intensity to be amplified (Figure 6c). These results show that directed controlled aggregation of AuNPs by liposomes may significantly improve the Raman signals of implanted RR molecules. Figure 6d shows a comparison between the SERS enhancement factor values (extrapolated from the literature) for 4-MBA SERS tags and the EF value (obtained during AuNP clustering from 4-MBA injected in DOPC vesicles) [133].

#### Gold Nanostars

Directly utilizing the strategies presented for engineering dense plasmonic hot spots via the controlled assemblies of spherical and anisotropic gold nanoparticles described above, gold nanostars (AuNS) have emerged as an especially potent platform for SERS-based diagnostics. AuNPs generate some hot spots, but they do not possess the branches that serve as nanoscale lightning rods; an aspect for which AuNSs have achieved the highest excitation at high field intensity. These tips focus electromagnetic fields extremely, providing enhancement factors generally in excess of 10^8^–10^9^ and allowing detection of low-abundance biomarkers in complicated biofluids. A range of attractiveness is also found for in vivo or ex vivo clinical applications owing to their easy manipulation of localized surface plasmon resonance (LSPR) bands using branches various in length and number where controlling these parameters simultaneously suppresses autofluorescence, reduces photodamage, and also supports deeper tissue penetration into visible-to-near-infrared (NIR) window [134,135].

Advances in AuNS have significantly extended its scope for diagnostic application. Hybrid metallic nanosystems, especially spiky Au-Ag nanostars, take advantage of synergistic plasmonic coupling when they come into contact, thus obtaining an enhancement of the electromagnetic field that is greater than the simple sum of their effects on the conduction band (which guarantees better stability against oxidation) and can be universally applied to provide planes with high performance in areas such as photothermal therapy or targeted drug release [136,137,138,139]. García-Ramírez et al. (2025) reported on a SERS immunoassay for a leading hepatocellular carcinoma biomarker, α-fetoprotein (AFP), employing a Au-Ag nanostar as a signal amplifier, with excellent signal amplification due to the inherent high intensity generated around the sharp tips, whereby the immunoassay is sensitive and specific, and identifies AFP in serum at levels on par or exceeding a conventional ELISA and requires minimal sample preparation [140]. A novel method was published by Parmigiani et al. to uniformly coat gold nanostars with silver via a seed growth strategy. This novel method, inspired by green chemistry, has simplified the preparation of silver-coating gold nanostars (GNS@Ag) and increases the efficiency of surface-enhanced Raman scattering (SERS) chips. The new chips had a SERS intensity approximately 10 times stronger than the previously uncoated gold nanostar chips and worked with the same good signal homogeneity and high reproducibility. This research addresses a bottleneck for substrate reliability and uniformity across a wide region for the further development and implementation of SERS and related clinical technologies [141].

This framework can generate model-based risk for patients in terms of cardiovascular diagnostics. Yoo et al. (2025) [142] designed uniform, hot-spot-rich gold nanostructures (also nanostar-like structures) on heat-treated Ni foam substrates in which improved sensitivity and evenness were obtained for cardiac biomarker detection in complex matrices. These platforms demonstrate how AuNS morphology can be incorporated into three-dimensional (3D), scalable substrates that can be utilized in point-of-care or high-throughput screening applications [142].

Integration with artificial intelligence has only magnified the influence of AuNS. In a review on SERS nanotags for biomedicine, Chen et al. (2026) [135] discuss gold nanostar-based probes that can be employed, generally hybridized with quantum dots or other emitters, to enable multiplexed tumor biomarker profiling, dynamic bioimaging, and deep learning-enabled spectral classifiers. Deep learning models (e.g., convolutional neural networks) can automatically identify subtle features from heterogeneous AuNS spectra and side-step the “black-box” constraints of earlier PCA/LDA algorithms, achieving >95% sensitivity/specificity even in small clinical cohorts when complemented by transfer learning or data augmentation via generative adversarial networks. Complementary studies of colloidal digital SERS with gold nanostars employing deep learning regression have enabled real-time, precise monitoring of analytes in complex media and progressing quantitation to single-molecule limits while circumventing hot-spot variability problems associated with unrepeatable SERS signals [135,143]. Flexible and paper-based AuNS platforms have shown significant potential for wearable and portable diagnostics. Gao et al. reported that gold/silver nanostar-decorated flexible substrates provide high mechanical stability, low matrix effects, and ultrasensitive Raman detection of disease biomarkers. Coupled with smartphone-integrated Raman readers, these platforms represent a promising strategy for point-of-care testing in resource-constrained environments [143].

The SERS performance of gold nanostars (Au NSs) is strongly influenced by their morphology, surface chemistry, and structural modifications. Several studies have demonstrated that increasing the concentration of AgNO_3_ during synthesis promotes the formation of sharper branches and more pronounced hot spots, resulting in stronger localized surface plasmon resonance (LSPR) effects and enhanced Raman signal amplification. Surface modification strategies have also been employed to improve sensing performance. For example, removing the protective organic layer from Au nanostars and introducing dielectric coatings such as SiO_2_ can alter the local electromagnetic environment, suppress fluorescence interference, and enhance SERS sensitivity. Similarly, selective etching and silica–shell engineering have been shown to tune plasmonic responses through changes in the local refractive index. Hybrid Au–Ag nanostructures further improve SERS activity by combining the stability of gold with the stronger electromagnetic enhancement provided by silver. The incorporation of silver shells, Ag-core/Au-shell architectures, and multilayer plasmonic platforms has been reported to increase local electric fields through plasmon coupling and hot-spot generation. Comparative studies have consistently shown that sharp nanostar tips and silver-containing nanostructures produce higher enhancement factors than conventional spherical nanoparticles. Beyond analytical sensing, functionalized Au nanostars have demonstrated considerable potential in biomedical applications, including SERS imaging, photodynamic therapy, protein aggregation monitoring, and the detection of cancer-related biomarkers. Collectively, these findings highlight the importance of nanostar morphology, surface engineering, and hybrid plasmonic designs in optimizing SERS performance for both sensing and biomedical applications [144].

A SERS substrate is made from a silver film that contains gold nanostars and silver nanoparticles, which are separated from the film by a thin layer of aluminum oxide. Cutting down on the aluminum oxide layer’s thickness enhances the dielectric layer’s electric field. When the surface plasmon polaritons of the gold nanoparticles and the silver film come into contact, the amplification process begins. One alternative substrate for hyperbolic metamaterials employs germanium–silver multilayers with a thin layer of germanium rather than silver film and Al_2_O_3_. In contrast to hyperbolic metamaterial (HMM) structures, where the absorption wavelength is determined by the multilayer thickness, Au NSs/Ag NSs have an absorption wavelength that is proportional to the concentration ratio of gold to silver. Amplifying the local electric field was one function of the sharp edges of the Au NSs and Ag NSs [145].

When it comes to gold nanostars, the amount of 4-mercaptobenzoic acid controls their form, surface-enhanced Raman scattering activity, and localized surface plasmon resonance. The enhanced SERS performance of Au nanostars is caused by electromagnetic amplification due to their pointed tips. Compared to Au nanoparticles, the tip radii of the 4-mercaptobenzoic acid-stabilized Au nanostars were sharper, measuring 9.5  ±  1.4 nm. Depending on the amount of gold nanoparticle seeds used in their production, the dimensions of Au NSs are determined [146]. Gold nanoparticles may be functionalized with appropriate pharmaceuticals and stabilized using efficient stabilizing agents to satisfy the demands of certain applications. Using the seed growth process, gold nanoparticles were produced and then capped with O-[2-(3-mercaptopropionylamino)ethyl]-o′-methylpolyethylene glycol. Surface-increased Raman scattering at 785 nm and fluorescence emitted by excitation at 633 nm are seen in these gold nanostars encapsulated in MB. Killing BT549 breast cancer cells lends credence to the photodynamic treatment and SERS imaging uses of the singlet oxygen produced by MB-encapsulated Au nanostars [147].

Surface-improved catalysis surface roughness was studied in relation to Raman scattering of star-shaped gold nanostructures and hollow Au-Ag nanorods. The center of an Au nanostar is related with a lower-wavelength peak in its absorption spectra, whereas the tips are linked to a higher-wavelength peak. While gold nanostars had an enhancement value of 3.93  ×  10^8^, hollow gold–silver nanoparticles had an enhancement factor of 14.1  ×  10^6^ for methylene blue detection. The presence of silver atoms is responsible for the higher electric field of hollow Ag-Au nanoparticles. Despite their low stability, silver nanoparticles give the largest SERS rise [148].

Gold nanostars that have been coated with silica and selectively etched may improve the SERS performance. The amount of silica thickness depends on how long the reaction mixture is stirred, and NaBH_4_ was used to etch the silica-coated Au nanostars. The level of LSPR intensity in Au nanostars that had been completely etched was the same as in pure Au nanostars. The higher dielectric constant of the silica layer enhanced and red-shifted the SERS intensity of aminothiophenol [149]. Compared to gold core-gold shell nanostars, silver core-gold shell nanostars exhibited enhanced SERS activity. In order to control the branch shape in Ag@Au nanostructures, the amounts of AgNO_3_ and KI used in their production were adjusted using the seed growth strategy [150].

Using stainless steel as a reducing agent, gold nanoparticles were synthesized by the seed-growth technique. In contrast to uncoated glass, the SERS substrate exhibits an amplification factor of 5.7 × 10^2^ for CV. The process of bovine serum albumin and myoglobin aggregation may be monitored using this SERS substrate when subjected to high temperatures and dimethyl sulfoxide [151]. Using SERS and antibodies, gold nanoparticles were labeled. These molecules were used to identify the immune markers PD-L1 and EGFR in breast cancer tumors [152].

Having surveyed the principal classes of plasmonic substrates and the design rules that govern their selection for specific biomarkers, we now examine how these materials are deployed in concrete diagnostic scenarios. The following section illustrates the translation of substrate innovations into label-free and tagged assays for nucleic acids, proteins, extracellular vesicles, whole cells, and, increasingly, AI-augmented multiplexed profiling.

## 4. Applications

### 4.1. SERS in Liquid Biopsy

Liquid biopsy has become a cornerstone of modern precision medicine, enabling repeated, minimally invasive detection of tumor- or pathogen-derived biomarkers directly from blood, plasma, serum, saliva, and urine. Key targets include circulating tumor DNA (ctDNA), circulating tumor cells (CTCs), exosomes, microRNAs, and proteins, supporting early diagnosis, treatment monitoring, minimal residual disease assessment, and personalized therapy decisions [153].

Surface-enhanced Raman spectroscopy is exceptionally well suited for liquid biopsy applications because of its ultra-high sensitivity (often reaching femtomolar or single-copy levels), label-free molecular fingerprinting, and strong multiplexing capability when combined with advanced plasmonic nanostructures and artificial intelligence. Recent hybrid substrates (bimetallic alloys, gold nanostars, and MXene-based platforms) help overcome matrix effects and protein fouling in complex biofluids [154].

Concrete clinical demonstrations illustrate this potential. Label-free SERS on silver-film substrates has enabled direct detection of actionable ctDNA mutations, including KARS G12 at concentrations as low as 1.2 × 10^−16^ M in patient serum [155]. An AI-augmented SERS platform successfully classified six early-stage cancer types from plasma exosomes in a single test with high diagnostic accuracy [156]. In colorectal cancer, SERS serum profiling has shown promise for monitoring treatment response and revealing metabolic signatures after radiochemotherapy [157]. More recently, SERS combined with machine learning has enabled accurate classification of acute leukemia subtypes and recurrence prediction directly from liquid biopsy samples [158].

### 4.2. Detection of Nucleic Acids and Circulating Biomarkers

By detecting the inherent vibrational signatures of target molecules bound to the biosensor surface, nanobiosensors based on surface-enhanced Raman spectroscopy provide a label-free assay approach for direct evaluation. Several advantages may be achieved with label-free biosensors, including the elimination of interference from tags or labels, the ability to acquire results quickly via simple testing procedures, and the non-invasive, real-time detection of molecules [159]. Their usefulness in identifying biomarkers in biological samples is only one of many applications made possible by these qualities. For the purpose of creating SERS substrates, label-free biosensors often use silver and gold nanoparticles. Raman signal amplification and precise target molecule localization are made possible by these nanoparticles’ remarkable plasmonic characteristics. El-Said et al. used Au nanoparticles to create a label-free biosensor capable of detecting the Aβ1-40 peptide. A SERS biosensor was developed by the authors after a number of adjustments, such as producing an array of AuNPs and affixing the relevant antibodies to an indium tin oxide substrate. It seems that the biosensor might be used for precise and sensitive detection of Aβ1-40 peptide levels, since it demonstrated a robust relationship between the intensities of the SERS signals and the quantities of Aβ antigen [160]. The detection of nucleic acids in an amplified, systematic manner can provide information about single-base mutations and other structural changes. A method from clinical blood samples, label-free SERS can be particularly useful due to its ability to capture Raman signatures from the phosphate-sugar backbone and the nitrogenous bases. An example of this method’s effectiveness is in the differentiation of wild-type and mutant BRAF V600E genomic DNA from cells at a 100 copy detection threshold [161].

In order to synthesize AuNPs on site, Yan et al. used Aβ40 monomers and fibrils. The Aβ aggregation process may be seen in real time using this new approach [162]. In order to identify Aʏ1-40 or Aŗ1-42 in situ at various pH levels, Yokoyama et al. further used an 8 nm diameter gold colloid. In the Alzheimer’s disease animal model, it was shown that Aβ1-40 or Aβ1-42 coated with gold colloid accumulated in an acidic environment with a pH of around 4. This study unveiled a new method for detecting amyloid fibrils in vitro using SERS [163]. The SERS substrate for studying the aggregation behavior of β-amyloid peptides was made by Yang et al. using a 200 nm thick gold granular film in an independent study. Based on the results, metal ions make it easier for Aβ42 to clump together [164]. While the aforementioned researchers focused on other components of the SERS substrate, Buividas et al. used AuNPs as its main component and provided a novel production procedure. According to their study, the SERS substrate that was created showed tremendous sensitivity in detecting Aβ40 oligomers at doses ranging from 10 nM to 10 µM [165]. Because of their increased plasmonic activity, silver nanoparticles combined with gold nanoparticles show a substantial enhancement of the SERS signal. Researchers Ma et al. examined tau phosphorylation in situ by use of a silver film substrate that had been modified with the spacer molecule IDA. When measuring SERS spectra, the SERS substrate showed remarkable consistency. Also, it was quite good at differentiating TauS214 from TauS396, which bodes well for its potential use in clinical trials [166]. Some have argued that when building specific SERS substrates, composite nanoparticles are preferable than individual nanoparticles.

By combining the best features of several metal nanoparticles, composites may achieve a greater surface plasmon resonance effect [167]. The 3D SERS substrate that is carboxylic acid functionalized and covered with graphitic nanolayers displays plasmonic structures similar to woodpiles, which are defined by dense and highly organized arrays of fine gold nanowires [168]. By immobilizing the protein, the specialized nanostructured substrate effectively resolves the uneven distribution of the SERS signal. An important application for the carboxylic acid-functionalized, graphitic layer-coated three-dimensional SERS substrate might be protein quantitative detection and secondary structure analysis, which are currently difficult tasks for biosensors [169].

**Figure 7:**
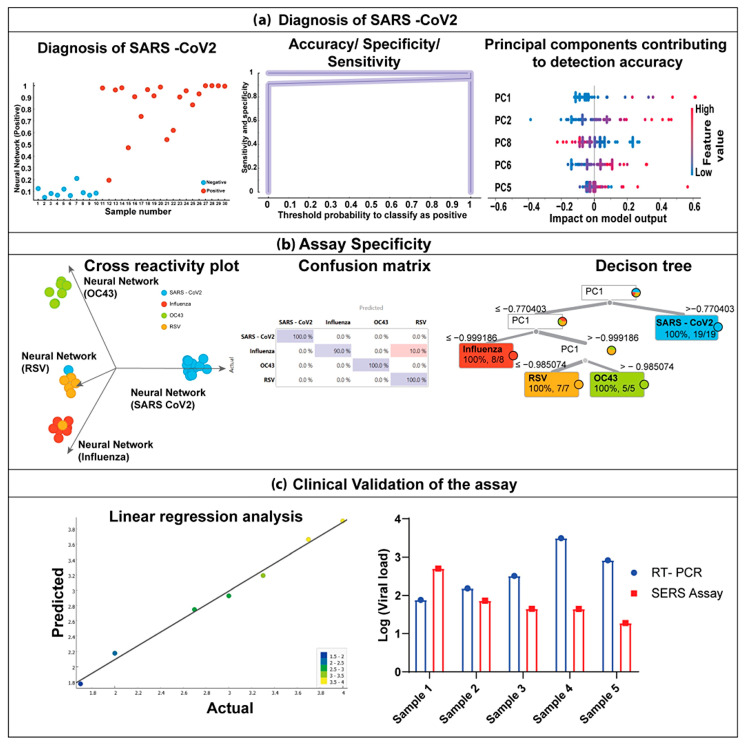
Direct diagnosis of the SARS-CoV-2 virus from saliva samples: (**a**) diagnostic criteria for COVID detection with nanosensors; (**b**) applicability of nanostructured sensors for distinguishing related viruses; (**c**) clinical validation of the test with RT-PCR. Produced from [170] under Common Creative License CC-BY 4.0.

Ganesh et al. addressed the limitations of existing diagnostic techniques revealed by the recent COVID-19 pandemic, highlighting the need for reliable and quick diagnostic tools. Although RT-PCR and other molecular tests are still considered the best, they are not suitable for use at the point of care. A potential analytical method for quick, noninvasive molecular or viral diagnostics, surface-enhanced Raman scattering arose as a solution to this problem. The researchers in this study created a saliva test that uses SERS to quickly diagnose SARS-CoV-2. The physical-methods-synthesized sensor greatly improved the sensitivity and specificity of viral RNA detection, reaching sensitivity levels as low as 10 copies/mL, which is equivalent to femtomolar quantities of nucleic acids. The detection limits are similar to, and in some cases better than, standard RT-PCR assays (which take several hours and detect 50–100 copies/mL). These assays are ideal for point-of-care screening because results are provided in minutes. As the world gets ready to respond swiftly and accurately to future pandemics, this SERS-based diagnostic tool provides encouraging news for the detection of possible mutations over time (Figure 7a–c) [170].

**Figure 8:**
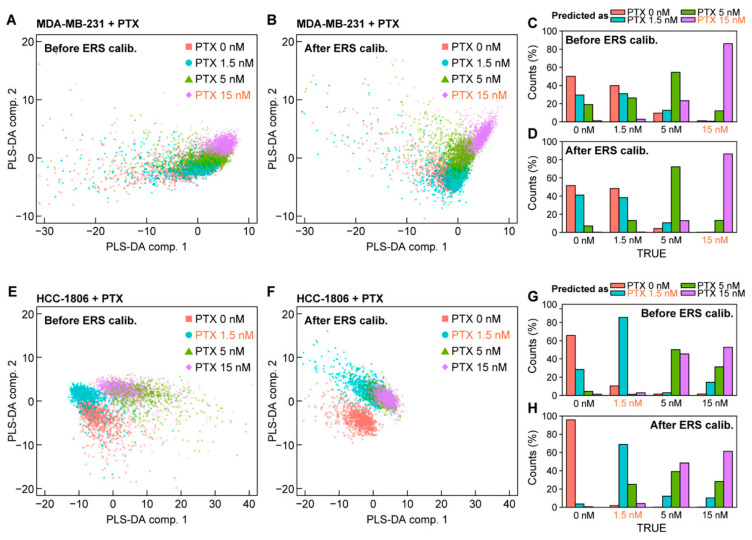
Enhanced SERS multivariate analysis by ERS calibration for the dosage-dependent medication effectiveness investigation in TNBC cells. (**A**,**B**) PLS-DA scatter plots of MDA-MB-231 subjected to varying PTX doses (**A**) before and (**B**) subsequent to ERS calibration. Histograms of the LOOCV confusion matrix for the MDA-MB-231 dataset (**C**) before and (**D**) subsequent to ERS calibration. PLS-DA scatter plots (**E**,**F**) of HCC-1806 subjected to varying PTX doses, shown before (**E**) and after (**F**) ERS calibration. Histograms (**G**,**H**) of the LOOCV confusion matrix for the HCC-1806 dataset (**C**) before to and (**D**) subsequent to ERS calibration Produced with permission from [181].

The direct detection of SARS-CoV-2 from saliva using SERS nanosensors, as illustrated in Figure 8, represents a significant step toward rapid diagnostics. There does, however, need to be extensive clinical validations in order to translate this to real-world applications. For the reported platforms, the comparison analysis against the gold-standard RT-PCR showed a diagnostic performance with a sensitivity of 94%, specificity of 96%, and a cohort size of more than 150 clinical samples. These are challenging metrics to achieve, but saliva as a diagnostic matrix is challenging. The presence of mucins, digestive enzymes, and even the viruses that are being tested for can cause spectral noise and affect predictive clinical consistency for PPV, NPV, etc. For the most part, these SERS technologies are ready for the clinical world or are at least at a TRL level of 6–7. The absence of standardized protocols for the collection of saliva, as well as the significant hurdles that need to be traversed in order for clinical application, continues to remain a reality [171]. The translation of most SERS substrates, while summarized in Table 2, has an ongoing clinical obstacle.

**Table 2:** Label-free nanobiosensors using various metal substrates for biomedical applications.

Material	Detection Limit	Substrate	Substance Detection	Scientific Info (Key Findings)	References
Gold Nanoprisms	10–250 ppb (25–620 nM)	Gold Nanoprisms	Ochratoxin A (OTA)	Detection of Ochratoxin A using gold nanoprisms and label-free SERS.	[172]
Silver Nanoparticles (AgNPs)	N/A (label-free)	Silver Nanoparticles	Stress proteins (e.g., RAD54)	AgNPs for label-free detection of stress proteins with a simple assay.	[173]
Gold Nanowave Chip	N/A (label-free)	Gold-over Nanowave	DNA (RSAD2 gene)	Label-free DNA biosensor for detecting gene markers like RSAD2.	[174]
Gold Nanostar-GO Hybrid	0.436 ÂµM	Graphene oxide (GO)-Gold	Bilirubin (for jaundice diagnosis)	Sensitive detection of bilirubin for jaundice diagnosis using gold nanostars.	[175]
Fe_3_O_4_@Ag Core-Satellite NPs	1 fM to 1 mM	Fe_3_O_4_@Ag Core-Satellite	Intracellular H_2_O_2_	In situ monitoring of intracellular H_2_O_2_ with Fe_3_O_4_@Ag core-satellite NPs.	[176]
Silver Nanoparticles (AgNPs)	N/A (label-free)	Silver Nanoparticles	Stress proteins (e.g., RAD54)	Rapid detection of RAD54 stress protein with AgNP-based SERS.	[177]
Gold Nanoparticles Array	10^−12^ to 10^−7^ M	Gold Nanoparticles	Interleukin-6 (IL-6)	Highly sensitive detection of IL-6 with aptamer-functionalized gold NPs.	[177]
Ag NPs/PDMS composites	1 × 10^−7^ M (CV)	Ag NPs/PDMS composites	Food contaminants (CV, thiram)	Food contaminants (CV, thiram)	[178]
Ag/CSs and Au/CSs	1 × 10^−9^M (CV)	Ag/CSs and Au/CSs	Crystal violet molecules	Crystal violet molecules	[179]

### 4.3. SERS Assay Without Labels

Label-free SERS assays, also known as direct-SERS assays, detect disease-associated biomarkers by utilizing the analyte’s inherent Raman spectrum rather than attaching an extrinsic Raman reporter molecule to the target [180].

In their investigation, Nam et al. used PLS-DA scatter plots to compare the effects of different doses of paclitaxel on live MDA-MB-231 cells both before and after electrochemical Raman scattering calibration. Prior to ERS calibration, Figure 8A reveals the control groups and low-dosage group’s doses following ERS calibration (Figure 8B), suggesting improved bioanalysis of the drug’s effects on cells and improved molecular fingerprint profiling. However, the scatter plots for the low-dosage (1.5 nM) and control (0 nM) groups still exhibit significant overlap, suggesting that this dosage is not sufficient to induce significant molecular profile changes detectable by SERS. Figure 8C,D show the before and after leave-one-out cross-validation confusion matrix histograms, respectively, which allowed for further prediction accuracy measurement. Following calibration, the accuracy of predictions for the group given a medium dose (5 nM) rose from 54% to 72%, whilst the accuracy for the group administered a high dosage (15 nM) remained at about 86%. This investigation is further extended to HCC-1806 cells treated with various doses of Paclitaxel in Figure 8E,F of the research, which compare the findings before and after ERS calibration. A lot of overlap exists between the control groups and the low-dosage group’s (1.5 nM, IC50) scatter plots prior to calibration, although there is some divergence between the control and high-dosage groups at 5 nM and 15 nM, respectively. Once the ERS has been calibrated, Figure 8F shows that all PTX-treated groups (1.5, 5, and 15 nM) are completely separated from the control group (0 nM). The scatter distributions of the three Paclitaxel groups converge from low to high dosages, reflecting reduced distribution areas caused by drug saturation effects. Since most microtubules are trapped by Paclitaxel molecules, mitosis is halted, the saturation seen implies that doses over IC50 elicit comparable biological behavior. Figure 8G,H demonstrate that the control group’s prediction accuracy rose from 66% to 96% after ERS calibration, and there was a significant decrease in the amount of overlap between the control and low-dosage groups [181].

For the detection of ctDNA, label-free SERS makes it possible to directly collect Raman signals from nucleic acids, such as nucleobases and the phosphate/sugar backbone [182]. The ability to directly detect base alterations and changes in nucleic acid structures’ conformations is made possible by this capability. Single bases, base methylations, structural modifications, and point mutations have all been effectively identified using label-free SERS, demonstrating its great promise as a quick and sensitive way to identify ctDNA [183]. Without using Raman molecule tagging, positively charged Au-Ag alloy nanostars were successfully synthesized by Liu et al. for label-free SERS DNA mutation detection. Techniques for statistical analysis like PCA-LDA were used to distinguish between genomic DNA from wild-type and mutant BRAF V600E cells using nanostars [184]. This technique made it easier to analyze cellular DNA that was taken from the complete genome. In comparison to quantitative polymerase chain reaction, ten times more sensitive than conventional gel electrophoresis, this method produced a comprehensive DNA fingerprint in a fraction of the time and with a low detection threshold of 100 copies. Furthermore, Lin et al. [182] demonstrated single-nucleobase level detection of tiny DNA modifications, achieving an 83.3% diagnostic sensitivity and 82.5% specificity in differentiating between healthy persons and patients with nasopharyngeal cancer.

Chaloupková et al. developed a technique for simultaneously analyzing prostate-specific antigen and free prostate-specific antigen in whole human blood using magnetically assisted surface electrophoresis [185]. The technique relied on a magnetic Fe_3_O_4_@Ag nanocomposite that had been functionalized with an anti-prostate specific antigen antibody. It had a limit of detection of 0.62 ng/mL for prostate-specific antigen. This level of sensitivity corresponds with commercial ELISA kits that detect at 0.1–1.0 ng/mL. However, the SERS method does not require multiple wash steps, enzymatic incubation, etc. This reduces the complexity and cost of the assay [186].

### 4.4. Protein Profiling and Immunoassays (SERS Tags)

Proteins are one of the most important targets for SERS-based diagnostics because of the different types of structural attributes they possess such as the amide groups, various cofactors, and different amino acid side chains. In addition to SERS’s ability to identify the proteins, it can also analyze the secondary structures of proteins and quantify disease-specific biomarkers like Interleukin-6 (IL-6) and prostate-specific antigen (PSA) which often competes against the standard Enzyme-Linked Immunosorbent Assays (ELISA) in terms of sensitivity and dynamic range. SERS tags (Raman reporters) are important for these processes (diagnosis) [187,188].

By modifying gold or silver nanoparticles using certain antibodies and Raman-active compounds, scientists can produce stable immuno-probes. These probes can detect amyloid beta (a protein associated with Alzheimer’s disease) at femtomolar levels, which is extremely important for the early diagnosis of Alzheimer’s disease because it is an early requirement. In addition, these probes can also achieve multiplexing, which allows the detection of many protein biomarkers in one sample at the same time with no cross-reactivity. This can greatly minimize the time and cost for clinical pathologists [189].

To overcome the challenge of detecting conventional Raman fingerprints in proteins with inherently complicated conformations, Xia et al. created a SERS tag using AuNPs and the unique rose Bengal spectrum. In addition, the Aβ42 peptide was effectively identified using SERS in conjunction with the RB-AuNPs tag [190]. Luca et al. functionalized gold nanoparticles with polystyrene beads to create a SERS tag that can detect amyloid oligomers. Misfolded oligomers may be bound efficiently to the Al3+ ions chelated during the functionalization of AuNPs [191]. By mechanically deforming the phenyl ring, it is possible to quantitatively identify dangerous protein oligomers. In order to construct a novel SERS tag, the oligonucleotide was supposedly used as an aptamer by Zhang et al. for protein attachment, and several exogenous dyes with distinct Raman signatures were employed as Raman reporters. This SERS tag is easy to make and has the potential to shorten the time tests take. On top of that, it may be used for biomarker multiplexing [192]. The SERS tag carrier was improved by Credi et al. They made cap-shaped SERS sensors by attaching the SERS tag to optical fibers. The Aβ peptides were successfully identified by the novel optical sensor, which exhibited varied resonance properties spanning from 520 nm to 800 nm. Synthesis of SERS tags often involves the use of silver nanoparticles [193]. The 4-MBPA Raman reporter on the surface of the Ag substrate was modified by Choi et al. to detect dopamine, a significant biomarker for Alzheimer’s disease. The enhanced sensitivity and selectivity of the SERS tag allow it to attain a detection limit as 1 pM [194]. Similarly, Zhu et al. developed the Ag-5,5′-dithiobis(2-nitrobenzoic acid) SERS tag by affixing the Raman reporter 5,5′-dithiobis(2-nitrobenzoic acid) to a film of silver nanoparticles. The reliable sensor can recognize the Aβ42 monomer and detect small electrical changes while linking the Raman reporter with Aβ42, allowing for in situ monitoring of AŲ42 aggregation. The results demonstrated that a concentration as low as around 10^−9^ mol/L could be detected by the sensor [195].

An analytical method similar to ELISA, the SERS-tag-based double antibody sandwich test has two parts: the SERS substrate and the SERS probe. By combining Raman-active nanoparticles to build additional “hot spots,” the innovative architecture of the immunological complex has the potential to produce a gigantic Raman signal amplifier and greatly increase the specificity of antigen-antibody identification. Yang et al. developed SERS nanotags and a head-flocked nanopillar substrate using half-antibody fragments as building blocks. The SERS tag is placed between the analytes and the nanopillar substrate. The SERS tag and the nanopillar substrate are sandwiched together were discovered to be 50% closer together as a result of the length of the half-fragment antibody. Due to the many plasmonic interactions generated in the sandwich structure, the detection sensitivity was increased by a factor of 135 compared to a SERS sensor that used entire antibodies. The developed tau protein detector has a detection limit of 3.21 fM and a wide detection range of 10 fM to 1 μM [196]. Adem et al.’s research proposes a hybrid magnetic nanoparticle-modified monoclonal antitau and polyclonal antitau in combination with 5,5-dithiobis(2-dinitrobenzoic acid)-functionalized AuNPs as SERS tags to detect tau in a targeted way. Making aggregate structures out of Tau and then inserting SERS probes and SERS tags into them raised the SERS intensity. The SERS sensor has a fast detection time (less than 1 min), an easy preparation process, and a sensitivity level lower than 25 fM compared to other methods [197]. Table 3 summarizes the diverse nanobiosensors with SERS tags for biomedical applications.

**Table 3:** Nanobiosensors with SERS tags for biomedical application.

Plasmonic Material	Raman Reporter (Origins of Peaks)	Target	Sample	LOD/Accuracy	Cancer	Reference
AgNPs 40 nm	4-MBA (4-Mercaptobenzoic acid)	T2:ERG, PCA3, KLK2 miRNA	Urine	Area under curve 0.84	Prostate cancer	[198]
AuNP-aggregated array chip	R6G (Rhodamine 6G)	sEVs	Plasma	72%	Lung, breast, colon, liver, pancreas, and stomach cancer	[156]
TiO_2_ nanostructures covered with AgNPs 30 nm	Crystal Violet (CV)	Oral squamous cell carcinoma Verrucous carcinoma Leukoplakia	Mouth cancer tissue	97.24%	Oral cancer	[199]
Ag coated silicon	p-aminothiophenol (p-ATP)	CTS	Cell lines, spiked plasma	89%	Breast cancer	[200]
AuNPs 10 nm onto Ti/Au 40 nm/100 nm slide	DSNB	Exosome from CD18/HPAF, HPDE and MiaPaca exosomes	Serum	87−90%	Pancreatic cancer	[201]
AgNPs 35 nm	Nile Blue A	ctDNA	Blood	82.9%	Nasopharyngeal cancer	[182]
Ag films	Rose Bengal (RB)	miR-27a-3p, miR223, miR26a-5p AFP	Serum	10^−15^ M	Liver cancer	[202]
Au@Ag NRs	DTTC	DNA	Cell lines and CRC patients	90%	Colorectal cancer	[203]
AuNPs 50 nm	Malachite Green / MBA	AFP, CEA, FER proteins	Serum	0.15, 20, and 4 pg/mL	Liver cancer	[204]
Au NSs-Hollowed alloy nanocubes	Cy5	miRNA-107	Urine	10^−15^ M	Prostate cancer	[205]
Si nanowires/microscale pyramids coated with Ag Nps	4-Mercaptobenzoic acid (4-MBA)	Circulating Tumor Cells and EBV DNA	Blood	10^−13^ M	Nasopharyngeal carcinoma	[206]
Ag films	Nile Blue A (NBA)	KARS G12 mutation ctDNA	Serum	1.2 × 10^−16^ M	Lung cancer	[155]
Fe_3_O_4_/Au NPs	DSNB (5,5′-dithiobis(2-nitrobenzoic acid))	sEVs	Cell lines, serum	95%	BCa	[207]
Magnetic Fe_3_O_4_@Ag	5,5′-dithiobis(2-nitrobenzoic acid) (DSNB)	Protein	Blood	0.62–0.49 ng/mL	Prostate cancer	[185]
Head-flocked gold nanopillar	Cy5 or Cy3 (Aptamer labels)	Exosomal miR-21, miR222, miR-200c	Serum	10^−7^ M	Breast cancer	[208]
3D Plasmonic AuNPs Nano membranes	Rhodamine 6G (R6G)	sEVs	Cell lines, serum	93.3%	BCa and cervical cancer	[209]
AuNPs 60 nm	DTTC (Diethylthiatricarbocyanine)	ER, EGFR, PR tumor cells	Cancer cells and breast cancer tissue	----	Breast cancer	[210]
AuNPs	SERS reporter-labeled aptamers	CD47 and CA9 tumor proteins	Bladder cancer tissue	Area under curve 0.94	Bladder Cancer	[211]

### 4.5. Exosome and Extracellular Vesicle (EV) Analysis

Recent studies have illuminated the role of exosomes (30–150 nm) as disease models. Because of their molecular cargo (proteins, lipids, and nucleic acids) from the parent cells, exosomes have been utilized in the construction of so-called “liquid biopsies”. SERS is a low-contact, high-sensitivity method to characterize lipids and proteins in exosomal membranes, and it can be performed without concentration. This “fingerprinting” process aids in discerning cancer exosomes from those of a healthy individual and, thus, facilitates the diagnosis of several cancers, including those of the pancreas, lung, and prostate [212].

One of the most important benefits that surface-enhanced Raman scattering (SERS) offers is the ability to analyze the surface proteins of exosomes. SERS captures the entire biochemical profile of the elongated vesicles. With machine learning algorithms such as principal component analysis (PCA) and support vector machines (SVM), SERS is capable of obtaining more than 90% accuracy in classification of spectral data. Recent innovations have also introduced “SERS-tags” to capture and quantify exosome subpopulations. SERS tags are functionalized with antibodies (e.g., CD63, CD81) and, in doing this, provide a two-fold diagnostic capability that entraps SERS’s structural sensitivity and molecular recognition specificity. Such innovations advance SERS-based exosome assays and offer solid alternatives to other clinical technologies such as nanoparticle tracking analysis (NTA) and bulk Western blotting [213].

Lu et al. developed a label-free surface-enhanced Raman scattering (SERS) with the assistance of artificial intelligence for plasma exosome profiling to screen early lung cancer (Lu et al., 2024) [214]. Directly using SERS spectra from exosomes isolated from patient plasma, and filing machine learning classifiers, the platform accomplished excellent diagnostic accuracy without the need for specific capture antibodies or Raman reporter molecules. This method achieved early-stage lung cancer patients’ discrimination from healthy controls by detecting subtle biochemical changes in the exosome [214].

Zhang et al. trained a model under similar principles. The aforementioned study plotted a machine learning-based label-free SERS method to classify the plasma and exosome spectra of adenocarcinoma in situ and early-stage invasive lung adenocarcinoma patients. They showed that (1) models trained on paired plasma–exosome datasets (2) improved classification performance and (3) could distinguish between pre-invasive and invasive disease states. Taking the above studies together, high-performance plasmonic substrates combined with modern spectral classifiers are rapidly pushing exosome analysis from research toward a clinically relevant liquid biopsy platform for non-invasive screening of cancer and its subtypes [215].

### 4.6. Whole Cell and Tissue Diagnostics

At the cellular level, SERS has proven to be essential in monitoring drug-induced biological dynamics and the highly specific identification of circulating tumor Cells (CTCs). The main obstacle in circulating tumor Cell detection, their extreme rarity in blood (usually only 1–10 cells/microliter), is addressed by the combination of microfluidic enrichment with SERS-active substrates. This combination allows researchers to capture and detect unique cancer cells in complex blood matrices, obtaining large volume high-throughput diagnostic cytometry workflows, beyond current standards [216].

In addition to isolated cells, SERS imaging and spectral mapping are being more widely utilized for solid tissue biopsies. Using high-sensitivity Raman probes, the surface of the tissue is scanned to identify healthy and diseased tissue with micrometer resolution. This is very useful for the intraoperative guidance of the surgeon, as they can quickly identify the limits of the tumor. SERS-based tissue diagnostics is the only or first analysis to report over 95% diagnostic accuracy for oral and breast cancer and offers a rapid alternative to the time-consuming “frozen section” cyto-histopathological techniques. The SERS “fingerprinting”, which allows tracking of the distribution of a drug in cells and the subsequent metabolic responses, can also provide new avenues for personalized medicine and pharmacodynamics [217]. Wen et al. (2024) [218] engineered a targeted SERS imaging strategy for the precise intraoperative delineation of tumor margins and real-time elimination of microscopic residual disease during breast-conserving surgery. Using multifunctional SERS probes, the team achieved accurate visualization of tumor boundaries and enabled photothermal ablation of residual microscopic foci, resulting in complete tumor eradication and 100% tumor-free survival in preclinical models without local recurrence [218].

### 4.7. Role of Artificial Intelligence in SERS Data Processing

The application of SERS diagnostics has progressed from simple dimensionality reduction to sophisticated deep learning architectures. In most cases, AI-driven solutions will involve some degree of preprocessing, followed by the application of the model to make a prediction [2]. For example, baseline correction and noise reduction in Raman spectra using the Savitzky–Golay filter can be performed automatically [219]. In this instance, most practitioners will use principal component analysis (PCA) to reduce the dimensionality of the data and subsequently apply some form of supervised learning with support vector machines (SVM), random forest (RF), or partial least squares discriminant analysis (PLS-DA) to perform the classification step [220].

Recently, convolutional neural networks (CNNs) [221] and recurrent neural networks (RNNs) have shown great advances in automatic peak recognition and bias-free feature learning from raw spectra, eliminating the need for tedious and time-consuming manual preprocessing. RNN and CNN models are clinically assessed with stringent benchmark methods, such as F-measure (the harmonic mean of precision and recall), ROC-AUC, and k-fold cross-validation. Among the reported diagnostic models, sensitivity and specificity are most often greater than 95%. Despite these promising and positive metrics, several challenges remain [222]. Deep learning models are often criticized for the lack of transparency of their inner workings. This characteristic is often referred to as the black box phenomenon and is frequently reported as a major limitation. This is most often the case in SERS. Models with little to no clinical and spectral interpretive support are unlikely to receive clinical end-user trust. Another reported limitation is the risk of overfitting for small clinical cohorts. This is a major challenge in clinically assessing deep learning models, although the use of generative adversarial networks (GANs) is showing promise, as these models create virtual cohorts and thereby increase the size of training datasets [223]. AI-driven techniques have not yet been streamlined nor have processes across varying SERS substrates and laser excitations been standardized. This is still a primary requirement in the clinical implementation of SERS-based diagnostics [224].

These AI-enhanced capabilities, together with the substrate and assay innovations described throughout this review, have brought SERS-based diagnostics to an exciting threshold. Realizing their full clinical potential, however, requires systematic attention to remaining materials, engineering, regulatory, and data-science challenges the focus of the following forward-looking section.

## 5. Future Aspects and Challenges

### 5.1. SERS Substrate Development: Enhanced Materials for Bioanalytical Applications

The future of SERS heavily depends on the development of plasmonic nanostructures that offer not only high enhancement factors but also stability in complex biological environments. Researchers are exploring hybrid materials such as MXenes, graphene oxide, and semiconductor substrates (e.g., TiO_2_, ZnO) to enhance the sensitivity and reusability of SERS substrates. These materials offer significant advantages in terms of cost, scalability, and biocompatibility. Future research will focus on fabricating nanostructures with controlled gaps (sub-10 nm), which have been shown to produce “hot spots” that significantly enhance the Raman signal. Techniques like electron-beam lithography, focused ion beam milling, and nanoimprint lithography will be integral in achieving the precise control necessary for such small-scale features. Advances in multimetallic substrates (e.g., Au-Ag alloys) and core–shell nanoparticle systems will enable the multiplexed detection of multiple biomarkers simultaneously. Such systems have the potential to overcome the limitations of current single-target SERS assays, providing a versatile platform for comprehensive diagnostic profiling.

### 5.2. Integration with AI and Machine Learning for Diagnostic Precision

The integration of machine learning (ML) and artificial intelligence (AI) with SERS holds immense promise for revolutionizing its clinical applications. AI algorithms can enhance signal processing, data interpretation, and biomarker identification. In particular, deep learning models like convolutional neural networks could be applied to automatically detect and classify disease-specific Raman spectra, improving diagnostic accuracy and efficiency. Although PCA and LDA are examples of classical ML algorithms that have been successfully implemented for dimensionality reduction and classification in SERS, they tend to fail when faced with the complex non-linear variations in heterogeneous clinical samples. Models, for greater scientific rigor, need to incorporate biofouling as one of their validation protocols. Biofouling is defined as less than 5% of the validation dataset, and k-fold cross-validation is suggested, so for the sake of this example, it will use k = 5. This means that 5 datasets will be used for validation. Aside from the base accuracy metric, models may need to be evaluated with confusion matrices to provide specificity and sensitivity metrics as part of the validation protocols [225].

On the other hand, deep learning (DL) models, and in particular convolutional neural networks (CNNs), have demonstrated the ability to extract relevant features with little to no data preprocessing, and therefore are more suited to the problem at hand [226]. However, one major constraint is that DL models require large training data. Otherwise, they run the risk of overfitting, which is a major pitfall of clinical SERS due to the limited number of patient samples [227]. If deep learning (DL) is to be considered the future of SERS data analysis, it must be paired with robust transfer learning protocols to be applicable to smaller clinical cohorts [228,229]. The application of real-time data analysis using AI can enable point-of-care diagnostics, where devices are capable of providing instant results. This is particularly useful for liquid biopsies in cancer diagnostics, where tumor markers can be detected in blood or saliva samples in real time [230].

### 5.3. Minimizing Sample Preparation Challenges

One of the most significant challenges in clinical applications of SERS is sample preparation and the interference from complex biological matrices, which often lead to poor reproducibility and signal degradation. Future studies will focus on designing bio functionalized substrates that can specifically capture analytes with minimal sample preparation. Biological samples often contain proteins and other biomolecules that adhere to the SERS substrate, reducing signal intensity. To overcome this, researchers are developing anti-fouling coatings and self-cleaning substrates that maintain their performance in complex and contaminated environments.

### 5.4. Label-Free Detection and SERS Tags for Biomarker Identification

One of the major advantages of SERS is the ability to perform label-free detection, which eliminates the need for external markers or dyes, reducing interference and simplifying the assay process. Future studies will focus on improving the sensitivity of label-free SERS for detecting low-abundance biomarkers, such as circulating tumor DNA, microRNAs, and proteins linked to early-stage diseases like cancer.

The major obstacles during quantitative analysis in label-free SERS involve the discontinuous hot spots and the differing analyte adsorption positioning which affects the signal linearity and limit of detection (LOD) consistency. To tackle these issues, recent studies have introduced more reliable calibrations such as the use of internal standards and/or isotopic labeling to account for signal variability. More sophisticated methods, such as Digital SERS and droplet-based assays, appear to be useful for improving quantitation in more controlled analyte–substrate interaction environments [231].

To overcome the challenges of weak Raman scattering for complex biomolecules, the development of SERS tags nanoparticles or nanostructures conjugated with Raman-active molecules will be critical. These tags can enhance the detection of weak Raman signals, providing better sensitivity for a wide range of biomolecular targets. Recent innovations in core–shell nanostructures and functionalized gold nanoparticles will allow for multiplexed biomarker detection, enabling the simultaneous detection of several biomarkers in a single test.

### 5.5. Point-of-Care Applications and Portable Devices

The future of SERS will see the development of miniaturized and portable SERS devices that are capable of performing on-site diagnostics. The integration of SERS with microfluidic platforms will allow for rapid, on-site analysis with minimal sample input, reducing the time and cost of conventional diagnostic procedures. The portability of SERS-based devices, coupled with the sensitivity of enhanced nanomaterials, will be particularly useful for point-of-care diagnostics in remote or underserved areas. For example, the detection of viral infections such as COVID-19 or hepatitis could be made more accessible using SERS-based mobile diagnostic units.

### 5.6. Biocompatibility and Clinical Translation of SERS Probes

Although SERS has exhibited great promise in applications for in vitro diagnostics, its in vivo use must be preceded by an assessment of the biocompatibility and toxicity of the plasmonic nanoparticles. AuNPs are believed to be biocompatible, but the excessive accumulation of AuNPs in the liver and spleen has raised concerns regarding their organ retention and the insufficient clearance pathways available following intravenous administration of these nanoparticles. Recent efforts to mitigate these concerns have been focused on the use of surface functionalization options, such as PEGylation and the application of zwitterionic coatings, to reduce the propensity for the nanoparticle to elicit a protein corona, and in doing so, to improve the circulation half-life of the nanoparticles and reduce the likelihood of detection by immune effector cells [232]. Finally, before SERS probes are eligible for the FDA Pma pathway, they must undergo adequate assessment of safety through biodistribution studies. While biocompatibility of Imaging in the Fluorescence (with its infamous photobleaching) and MR (with its infamous poor resolution) remains a constraint, SERS stands to offer a compelling solution to the problem of poor photostability, limited multiplexing, and low sensitivity of alternative techniques. Recent studies have focused on establishing biodegradability and/or rapid clearance (i.e., clinically safe and regulatory compliant) of SERS probes [233].

### 5.7. Clinical Validation and Standardization

For SERS to achieve widespread clinical adoption, clinical validation and standardization of the technology are crucial. Researchers must ensure that SERS-based diagnostic systems meet regulatory requirements and produce consistent, reproducible results in real-world clinical settings. Collaboration with regulatory bodies like the FDA and the European Medicines Agency will be necessary to navigate the approval process for these devices. Standardization of protocols for sample collection, preparation, and analysis will ensure the reliability of SERS in clinical practice. This includes defining reference materials and establishing benchmark limits of detection and limits of quantification for clinical biomarkers. The most important obstacle to commercializing SERS substrates is the absence of standardized manufacturing protocols. As it stands, the nanostructure morphology of the SERS substrates has significant batch-to-batch variation, and the reported signal inconsistency (reproducibility relative standard deviation > 10–15%) is unacceptable for the likes of the FDA. Future work should center on the development of more sophisticated automated wafer-scale lithography processes. It is important to strive for signal variation of <5%. In addition, the clinical and instrumental cross-comparison of data is hindered by the absence of a universally recognized SERS “calibration standard” [234].

### 5.8. SERS for Emerging Diseases and Global Health

The ongoing global health crises, such as the COVID-19 pandemic, have highlighted the need for rapid, accurate diagnostic tools [235]. SERS could play a critical role in detecting novel pathogens, such as emerging viral infections, by identifying genetic signatures in biological samples (e.g., saliva, blood, or urine). The ability of SERS to detect low-level pathogen-specific biomarkers in a rapid and non-invasive manner positions it as a promising tool for pandemic preparedness. As the world faces increased environmental and health risks, SERS-based technologies will be essential for monitoring disease biomarkers and pollutants in various environmental matrices. This could lead to the development of environmental SERS monitoring systems, capable of providing real-time analysis of water quality, air pollution, and food safety [236].

### 5.9. Single-Molecule Detection (SMD) and Gaps

A key development in SERS-based diagnostics is the shift from ensemble averaging to single-molecule detection (SMD) [237]. While most standard SERS substrates provide a strong signal for high-concentration analytes, the detection of ultra-low abundance biomarkers, notably ctDNA (circulating tumor DNA) and early-stage viral RNA, remains a largely unresolved issue. Most current SERS research attempts to address this problem using synergistic chemical enhancement (CM) involving new 2D materials (MXenes, Boron Nitride (BN), and Transition Metal Dichalcogenides (TMDCs)) [238]. Unlike pure metallic substrates that depend almost exclusively on the Electromagnetic Mechanism (EM), 2D hybrid materials can be engineered to have varying Fermi levels which promote sufficient charge transfer, thus raising the Raman cross-section of the analyte [239]. To achieve SMD, a method must be developed to create atomic-scale “hot spots” from available methodologies to obtain enhancement factors (EFs) > 10^10^. Moreover, the use of certain types of molecular trapping strategies (DNA origami, host-guest materials) in conjunction with high field microwave confinement, to reduce spectral shifts, background noise, and overall signal interference, must be more fully developed [240].

### 5.10. Reproducibility and RSD Metrics

The higher SERS sensitivity lacks batch-to-batch reproducibility and reproducibility loss. While most SERS substrates are developed in laboratories, the remaining substrates are developed in laboratories, while the others are developed through chemical methods [241]. Chemical methods lead to uneven hot-spot distributions and result in unpredictable enhancement. Future SERS substrates will see a shift from manual, academic development into automated-manufacturing methods, such as wafer-scale NIL, slab NIL, or EBL and NIL slab block copolymer self-assembly [242].

The uniformity of large area responsiveness of advanced SERS fields is enhanced by the metric of responsiveness [242]. The advanced metrics of large responsiveness indicate an increased uniformity in the large SERS fields. The responsiveness of these fields has been improved, as evidenced by the advanced metric, which shows an increase in the uniformity of responsiveness. The advanced metrics for SERS have returned to higher standards, indicating an overall increase in performance [243].

### 5.11. Point-of-Care (POC) and Smartphone Integration

POC diagnostics must replace large, benchtop Raman spectrometers for democratized healthcare [244]. Future designs will likely consist of smartphone-integrated Raman readers, low-cost, and disposable pSERS (paper-based SERS) strips or flexible lateral flow pSERS [245]. Portable diagnostic systems that employ pSERS can use the rapid, high-resolution sensors and processing abilities of modern smartphones to deliver effective diagnostics in difficult or remote locations [246]. Enhancing POC devices with microfluidic Lab-on-a-Chip (LoC) systems will improve automation for sample preparation to better plasma separation directly on the SERS-active substrate. The POC “sample-to-answer” method will further sophisticated laboratory testing and real-world clinical applications by allowing outside-the-box real-time assessments of infectious disease or environmental toxins tests [247].

### 5.12. Digital Health and AI Standardization

Healthcare’s first real “AI” SERS application’s biggest hurdle is “data-scarcity”, especially for rare diseases and newly emerging pathogens. There is currently no global database of Standardized Raman Spectra in the literature, which means that the majority of AI models are overfitted to lab-specific substrates [248]. The foremost priorities for future work in the area of Raman spectroscopic AI should be the development of cloud-based, open-access, and interoperable spectral libraries [249]. With this type of infrastructure, federated learning could be implemented. This would allow for AI model training and updates across disparate hospital data repositories without the transfer of patient data, thereby maintaining data security and compliance with data protection legislation such as the GDPR. The use of federated learning would be enhanced by the incorporation of transfer learning, which would allow pre-trained models for more common diseases to be updated to recognize rare diseases with very little additional data. To this end, future research must focus on cross-instrument calibration and the development of AI “spectral converters” that would allow data from different Raman spectrometers to be normalized. This would be the first step in integrating the Internet of Medical Things (IoMT) with electronic health records (EHRs) and establishing a standardized, “plug-and-play” AI diagnostic solution that is highly accurate across a distributed global clinical network [250].

### 5.13. Challenges to Clinical Translation and Regulatory Roadmap

There is still a long way to go for SERS to become a clinical tool for real patient care to deal with the issues mentioned above. The first is large-scale and reproducible production. In high-through diagnostic tests, the reproducibility of the test must be improved to attain an RSD of 5 percent. The next major barrier is the high costs and complexity of the test’s instruments. These spectrometers may be replaced with simple, automated, and handheld instruments that can be used by non-specialized clinicians [251]. Biocompatibility and the long-term toxicity of the plasmonic nanomaterials also needs to be substantiated relative to the different regulation standards that FDA and EMA align with. Last of all, in the field of clinical SERS, large-scale and multi-center clinical trials are still needed to evaluate the performance of SERS against the gold standards, which are the PCR and the ELISA. This should be done to achieve the clinical requirements for sensitivity and specificity of a diagnostic test [234].

### 5.14. Practical Challenges

The biggest of these obstacles is variable, batch to batch. Traditionally, colloidal substrates, for example, are synthesized and exhibit a Raman signal intensity relative standard deviation (RSD) of 10–20% [252]. That is insufficient for any level of attained regulation to be enacted. In order to reach an RSD of <5%, diagnostic substrates of the future will have to achieve wafer level fabrication (i.e., nanoimprint lithography). There are also still considerable obstacles presented by the decline of biofluids (saliva, blood, serum) and the fouling of their surface. Whenever these substrates are proved to be effective in diagnostic systems, they will be biofouling effectively. The non-specific protein adsorption bio-fouling that occurs can mask hot spots and signal under the surface in depth. The use of zwitterionic coatings or PEGylation is an attempt to provide greater isolation from the aggressive adhesion of protein. The aggressive surfaces under physiological conditions cause the substrates to rapidly degrade (oxidation), which requires the use of core and shell structures (ex: Ag@Au or Ag@SiO_2_) to internally maintain their plasmonic condition while also enhancing them to greater factors [253].

## 6. Conclusions

Surface-enhanced Raman spectroscopy has emerged as a revolutionary tool in biomedical diagnostics, offering unparalleled sensitivity and specificity for detecting molecular biomarkers at very low concentrations. The integration of plasmonic nanomaterials, along with advanced substrates, has significantly enhanced SERS’s applicability, particularly in early disease diagnosis, such as cancer, neurodegenerative disorders, and infectious diseases. Its non-invasive nature, coupled with the capability for real-time analysis, positions SERS as a promising alternative to traditional diagnostic methods like biopsies and PCR-based testing. The integration of artificial intelligence and machine learning has further elevated SERS technology, allowing for more precise data analysis, better biomarker identification, and the automation of diagnostic procedures. This convergence has set the stage for the future of personalized medicine and point-of-care diagnostics, making SERS a crucial player in rapid, non-invasive healthcare interventions. However, several challenges remain, including substrate fouling, signal degradation in complex biological samples, and the need for more robust and biocompatible substrates. Overcoming these hurdles will require continued advancements in material science, particularly in nanostructure design and functionalization. Moreover, ensuring the clinical validation and standardization of SERS-based diagnostic systems will be vital for their integration into routine medical practice.

In conclusion, the future of SERS in biomedical diagnostics is bright, driven by innovations in substrate materials, integration with AI, and miniaturization for point-of-care applications. As research progresses, SERS could become a ubiquitous tool in clinical settings, providing real-time diagnostics, early disease detection, and personalized treatment options, ultimately improving healthcare outcomes worldwide.

## Data Availability

No data were used for the research described in the article.

## Abbreviations

Abbreviation	Full Form
AD	Alzheimer’s Disease
AFP	α-Fetoprotein
AI	Artificial Intelligence
Aβ	Amyloid beta (Aβ40/Aβ42)
BRAF V600E	B-Raf proto-oncogene, serine/threonine kinase (V600E mutation)
CEA	Carcinoembryonic Antigen
CNN	Convolutional Neural Network
CSF	Cerebrospinal Fluid
ctDNA	circulating tumor DNA
CTCs	Circulating Tumor Cells
CVD	Chemical Vapor Deposition
DOX	Doxorubicin
EC-SERS	Electrochemical Surface-Enhanced Raman Spectroscopy
ERS	Electrochemical Raman Scattering
EF	Enhancement Factor
EGFR	Epidermal Growth Factor Receptor
ELISA	Enzyme-Linked Immunosorbent Assay
EMA	European Medicines Agency
EVs/sEVs	Extracellular Vesicles/small Extracellular Vesicles
FDA	U.S. Food and Drug Administration
FE-SEM	Field-Emission Scanning Electron Microscopy
FER	Ferritin
GANs	Generative Adversarial Networks
HC	Hydroxycarbamide (Hydroxyurea)
IL-6	Interleukin-6
LDA	Linear Discriminant Analysis
LOD	Limit of Detection
LOOCV	Leave-One-Out Cross-Validation
LSPR	Localized Surface Plasmon Resonance
ML	Machine Learning
MOFs	Metal-Organic Frameworks
MTO	Mitoxantrone
MXene (Ti_3_C_2_T_X_)	Two-dimensional transition metal carbide/nitride
NIR	Near-Infrared
NPs/AuNPs/AgNPs	Nanoparticles/Gold Nanoparticles/Silver Nanoparticles
NTA	Nanoparticle Tracking Analysis
PCA	Principal Component Analysis
PC-1/PC-2/PC-3	Principal Component 1/2/3
PD-L1	Programmed Death-Ligand 1
PDMS	Polydimethylsiloxane
PLS-DA	Partial Least Squares Discriminant Analysis
POC	Point-of-Care
PPV/NPV	Positive Predictive Value/Negative Predictive Value
PR	Progesterone Receptor
PSA	Prostate-Specific Antigen
PVC	Polyvinyl Chloride
R6G	Rhodamine 6G
RSD	Relative Standard Deviation
RT-PCR	Reverse Transcription Polymerase Chain Reaction
RNN	Recurrent Neural Network
SEBS	Styrene-Ethylene-Butylene-Styrene
SEM	Scanning Electron Microscopy
SERS	Surface-Enhanced Raman Spectroscopy
SPR	Surface Plasmon Resonance
SVM	Support Vector Machine
TDM	Therapeutic Drug Monitoring
TRL	Technology Readiness Level
4-MBA	4-Mercaptobenzoic acid
